# Interleukin‐4 receptor alpha signaling regulates monocyte homeostasis

**DOI:** 10.1096/fj.202101672RR

**Published:** 2022-09-05

**Authors:** Patrick Haider, Julia B. Kral‐Pointner, Manuel Salzmann, Florian Moik, Sonja Bleichert, Waltraud C. Schrottmaier, Christoph Kaun, Mira Brekalo, Michael B. Fischer, Walter S. Speidl, Christian Hengstenberg, Bruno K. Podesser, Kurt Huber, Ingrid Pabinger, Sylvia Knapp, Frank Brombacher, Christine Brostjan, Cihan Ay, Johann Wojta, Philipp J. Hohensinner

**Affiliations:** ^1^ Division of Cardiology, Department of Internal Medicine II Medical University of Vienna Vienna Austria; ^2^ Ludwig Boltzmann Institute for Cardiovascular Research Vienna Austria; ^3^ Division of Haematology and Haemostaseology, Comprehensive Cancer Center, Department of Internal Medicine I Medical University of Vienna Vienna Austria; ^4^ Division of Vascular Surgery, Department of General Surgery Medical University of Vienna Vienna Austria; ^5^ Institute of Vascular Biology and Thrombosis Research Medical University of Vienna Vienna Austria; ^6^ Department of Blood Group Serology and Transfusion Medicine Medical University of Vienna Vienna Austria; ^7^ Department of Biomedical Research Danube University Krems Krems Austria; ^8^ Center for Biomedical Research Medical University of Vienna Vienna Austria; ^9^ 3rd Department of Medicine, Cardiology and Intensive Care Medicine Wilhelminenhospital Vienna Austria; ^10^ Medical Faculty Sigmund Freud University Vienna Austria; ^11^ Laboratory of Infection Biology, Department of Internal Medicine I Medical University of Vienna Vienna Austria; ^12^ Institute of Infectious Disease and Molecular Medicine International Center for Genetic and Biotechnology Cape Town Component & University of Cape Town Cape Town South Africa; ^13^ Core Facilities Medical University of Vienna Vienna Austria

**Keywords:** homeostasis, immunity, innate, interleukin‐4, monocytes, receptors, signal transduction

## Abstract

Interleukin‐4 (IL‐4) and its receptors (IL‐4R) promote the proliferation and polarization of macrophages. However, it is unknown if IL‐4R also influences monocyte homeostasis and if steady state IL‐4 levels are sufficient to affect monocytes. Employing full IL‐4 receptor alpha knockout mice (IL‐4Rα^−/−^) and mice with a myeloid‐specific deletion of IL‐4Rα (IL‐4Rα^f/f^ LysM^cre^), we show that IL‐4 acts as a homeostatic factor regulating circulating monocyte numbers. In the absence of IL‐4Rα, murine monocytes in blood were reduced by 50% without altering monocytopoiesis in the bone marrow. This reduction was accompanied by a decrease in monocyte‐derived inflammatory cytokines in the plasma. RNA sequencing analysis and immunohistochemical staining of splenic monocytes revealed changes in mRNA and protein levels of anti‐apoptotic factors including *BIRC6* in IL‐4Rα^−/−^ knockout animals. Furthermore, assessment of monocyte lifespan in vivo measuring BrdU^+^ cells revealed that the lifespan of circulating monocytes was reduced by 55% in IL‐4Rα^−/−^ mice, whereas subcutaneously applied IL‐4 prolonged it by 75%. Treatment of human monocytes with IL‐4 reduced the amount of dying monocytes in vitro. Furthermore, IL‐4 stimulation reduced the phosphorylation of proteins involved in the apoptosis pathway, including the phosphorylation of the NFκBp65 protein. In a cohort of human patients, serum IL‐4 levels were significantly associated with monocyte counts. In a sterile peritonitis model, reduced monocyte counts resulted in an attenuated recruitment of monocytes upon inflammatory stimulation in IL‐4Rα^f/f^ LysM^cre^ mice without changes in overall migratory function. Thus, we identified a homeostatic role of IL‐4Rα in regulating the lifespan of monocytes in vivo.

**Abbreviations**
BrdU: bromodeoxyuridine
CXCR4: CXC‐motif chemokine receptor 4
MCP‐1: monocyte chemoattractant protein‐1
M‐CSF: macrophage colony‐stimulating factor
sTNF‐RI: soluble tumor necrosis factor receptor‐I

## 1 INTRODUCTION

Interleukin (IL)‐4, produced by cells such as mast cells, granulocytes or ILC2 cells, is, like IL‐13, a key cytokine modulating the immune system to propagate an expansion of T_helper_‐2 cells.^1^ Furthermore, both cytokines lead to the polarization of macrophages to an alternatively activated state associated with wound healing, helminth infections, and allergic disorders.^2^, ^3^

IL‐4 binds with high affinity to the IL‐4 receptor alpha chain (IL‐4Rα, CD124) together with the common gamma chain (γ_c_, CD132) or with the IL‐13 receptor alpha 1 chain (IL‐13Rα1, CD126), whereas IL‐13 binds preferably to the IL‐13Rα1 chain, which then forms a heterodimer with IL‐4Rα. Myeloid cells express both types of IL‐4 receptors, “Type I” receptors (IL‐4Rα, γ_c_) as well as “Type II” receptors (IL‐4Rα, IL13Rα1).^4^, ^5^ While Type II receptor activation mainly leads to the phosphorylation of STAT6,^6^ Type I receptors further recruit and activate insulin receptor substrate 2 molecules, leading to the downstream activation of phosphoinositide 3‐kinase and Ras GTPase pathways.^7^

Recently, IL‐4 was shown to be an alternative proliferation factor for tissue macrophage populations independent of macrophage colony‐stimulating factor (M‐CSF).^8^ This expanded role of IL‐4 beyond inflammation as a homeostatic factor led us to hypothesize, that IL‐4 signaling not only affects macrophages, but also monocytes, as these cells share a common myeloid progenitor.

By making use of a full and a myeloid‐specific IL‐4Rα knockout mouse line, we found that IL‐4 receptor expression during homeostatic conditions determined the amount of circulating monocytes by prolonging their lifespan as well as by reducing the induction of cell death and subsequent removal in the spleen. These findings were further supported by an exploratory analysis in a human cohort of 458 individuals. IL‐4 levels in serum were significantly associated with the blood level of monocytes in this cohort. Furthermore, the recruitment of monocytes to sites of inflammation was diminished in IL‐4Rα‐deficient mice with reduced circulating monocyte amounts, although IL‐4 was not found to be chemotactic to monocytes. Thus, our study identified a previously unknown role of IL‐4Rα signaling as regulator of monocytes' amount and lifespan in homeostasis.

## 2 MATERIAL AND METHODS

### 2.1 Study approvals and ethics declarations

All animal experiments were performed in accordance to the NIH Guide for the Care and Use of Laboratory Animals and were approved by the animal ethics board of the Medical University of Vienna and the Austrian Federal Ministry of Education, Science and Research (BMBWF 2020‐0.073.943).

Patients were included after providing their written informed consent for study participation. The study was conducted in accordance with the Declaration of Helsinki and approved by the ethics committee of the Medical University of Vienna (EK 126/2003).

Whole blood was collected from healthy human donors after written informed consent was given. All studies were performed according to recommendations of the ethical board of the Medical University of Vienna (EK 1575/2014).

### 2.2 Mice

C57BL/6J mice were bred in house; IL‐4Rα^−/−^ mice,^9^ IL‐4R^f(loxP)/f(loxP)^ mice^10^ and their respective littermates were provided by Frank Brombacher; LysM^cre^ mice were provided by Sylvia Knapp. Animals were kept under specific pathogen free conditions. Room temperature and humidity were kept constant at 22° ± 1°C and 50%–60% humidity, and a 12/12 h light/dark cycle was used. Animals were kept in groups of 5 in type IIL Polysulfon cages. They were fed with standard chow and provided with autoclaved drinking water ad libitum. The animal floors were secured 24 h a day and animals were checked daily by the care takers or researchers.

### 2.3 Human subjects

Patient data as well as blood monocyte counts and serum IL‐4 levels were obtained from the Vienna CATS study, a large‐scale, prospective, observational cohort study conducted at the Medical University of Vienna, Austria. Specifics on study design and characteristics were reported in detail previously.^11^ Of note, the Vienna CATS study included only cancer patients with newly diagnosed cancer or progression of disease after complete or partial remission who have not recently received chemotherapy (within the last 3 months), radiotherapy, and surgery (within the last 2 weeks). For this exploratory analysis, a sub‐cohort of patients was selected from the overall study population based on the availability of baseline monocyte counts and serum IL‐4 levels, excluding patients with hematologic and cerebral malignancies due to their specific nature of the disease.

### 2.4 Primary human monocyte culture

Peripheral blood mononuclear cells (PBMCs) were collected by density gradient centrifugation as published previously.^12^ Monocytes were then isolated from the PBMCs by negative selection using EasySep Human Monocyte Enrichment Kit without CD16 Depletion (Stemcell Technologies, Vancouver, British Columbia, Canada) following the manufactures' protocol. In total, monocytes from 12 male and 7 female individual human donors were included into this study.

### 2.5 Genotyping of mice

Each animal, except the C57BL/6J wild‐type mice, was genotyped before the experiment. Therefore, tissue was collected at time of toe clipping (neonatal mice at the age of 5–7 days) and used for genotyping. DNA was extracted using the Maxwell 16 Mouse Tail DNA Purification Kit on a Maxwell robot (both Promega, Madison, WI, USA). PCR was performed using GoTaq G2 Green Master Mix (Promega, Madison, WI, USA) as well as the following primers: IL‐4Rα P1 (ggctgcagacctggaataacc), IL‐4Rα P2 (cctttgagaactgcgggct), IL‐4Rα EXON 8R (ctcggcgcactgacccatct), IL‐4Rα EXON 7F (tgacctacaaggaacccaggc), IL‐4Rα INTRON 6R (gtttcctcctaccgctgatt), LysMcre425 (cccagaaatgccagattacg), LysMcre426 (cttgggctgccagaatttctc), LysMcre427 (ttacagtcggccaggctgac). Amplified PCR products were then added together with a GeneRuler 100 bp Plus DNA Ladder (Thermo Fisher Scientific, Waltham, MA, USA) onto E‐Gels 2% with Sybr Safe (Thermo Fisher Scientific, Waltham, MA, USA) and electrophoresis was performed in an E‐Gel Power Snap Electrophoresis Device (Thermo Fisher Scientific, Waltham, MA, USA).

### 2.6 Flow cytometry

For flow cytometric staining, cells were resuspended in phosphate buffered saline solution (PBS), pH 7.4, containing 2% fetal bovine serum (FBS, MP Biomedicals, Santa Ana, CA, USA), and unspecific antibody binding was blocked using anti‐human/mouse TruStain FcX (both Biolegend, San Diego, CA, USA), respectively. Premixed 2x antibody cocktails were added 1:1 to the samples and cells were stained for 15 min at room temperature in the dark. Cells were stained with mixtures of the following antibodies diluted 1:100 in PBS: CD45‐BV650 (30‐F11), CD11b‐PerCP (M1/70), CD11b‐BV711 (M1/70), Ly6G‐APC (1A8), Ly6G‐Alexa488 (1A8), Ly6G‐Alexa700 (1A8), CD115‐PE (AFS98), Ly6C‐BV605 (HK1.4), Ly6C‐PacificBlue (HK1.4), CD3e‐Alexa488 (145‐2C11), B220‐BV421 (RA3‐6B2), F4/80‐FITC (BM8), F4/80‐PE/Cy7 (BM8), CD19‐Alexa488 (6D5), NK1.1‐Alexa488 (PK136), CD117‐APC/Cy7 (2B8), CD135‐BV421 (A2F10) and CXCR4‐APC (L276F12, all Biolegend, San Diego, CA, USA). For apoptosis assessment, cells were stained in 1× AnnexinV binding buffer (eBioscience, San Diego, CA, USA) with AnnexinV‐APC (eBioscience, San Diego, CA, USA) and 7‐AAD (Biolegend, San Diego, CA, USA) following the manufacturers' instructions. All samples were analyzed on an Attune NxT flow cytometer with 3 laser (BRV) configuration (Thermo Fisher Scientific, Waltham, MA, USA). The absolute amount of cells per μl blood or per gram tissue was calculated from the flow cytometric data.

### 2.7 Protein quantification

Cytokine screening was performed in IL‐4Rα knockout mice and wild‐type littermates using the 40 Targets Mouse Inflammation Antibody Array (Abcam, Cambridge, Cambridgeshire, United Kingdom). Validation of cytokine levels was performed in IL‐4Rα^−/−^, IL‐4Rα^f/f^LysM^cre^ mice and their littermates as well as C57BL/6J mice treated with vehicle or IL‐4, using specific ELISAs for the significantly regulated cytokines MCP‐1 (Mouse CCL2/JE/MCP‐1 Quantikine ELISA kit, R&D Systems, Minneapolis, MN, USA), IL‐1α (IL‐1 alpha Mouse Uncoated ELISA kit, Thermo Fisher Scientific, Waltham, MA, USA), IL‐6 (IL‐6 Mouse Uncoated ELISA kit, Thermo Fisher Scientific, Waltham, MA, USA), and sTNF‐RI (Mouse/Rat RNF RI/TNFRSF1A Quantikine ELISA kit, R&D Systems, Minneapolis, MN, USA). Differences in the phosphorylation of proteins involved in apoptotic pathways in human monocytes cultured for 24 h either untreated or with 100 ng/ml IL‐4 (Biolegend, San Diego, CA, USA) in serum‐depleted medium were assessed using the RayBio C‐Series Human Apoptosis Signaling Pathway Array (RayBiotech, Peachtree Corners, GA, USA).The increase in IL‐4 levels in the plasma of C57BL/6J mice treated with IL‐4 compared to vehicle was verified by an IL‐4 ELISA (Mouse IL‐4 Quantikine ELISA kit, R&D Systems, Minneapolis, MN, USA).

### 2.8 RNA sequencing and data analysis

Splenic monocytes were isolated from single cell suspension of spleens using the EasySep Mouse Monocyte Isolation Kit (Stemcell Technologies, Vancouver, British Columbia, Canada). RNA was isolated using the Maxwell RSC simplyRNA tissue kit (Promega, Madison, WI, USA) on a Maxwell RSC isolation robot (Promega, Madison, WI, USA). RNA sequencing was performed at the Core Facilities of the Medical University of Vienna. Sequencing libraries were prepared using the NEBNext Poly(A) mRNA Magnetic Isolation Module and the NEBNext Ultra™ II Directional RNA Library Prep Kit for Illumina according to the manufacturers' protocols (New England Biolabs, Ipswich, MA, USA). Libraries were QC checked on a Bioanalyzer 2100 (Agilent Technologies, Santa Clara, CA, USA) using a High Sensitivity DNA Kit (Agilent Technologies, Santa Clara, CA, USA) for correct insert size and quantified using Qubit dsDNA HS Assay (Thermo Fisher Scientific, Waltham, MA, USA). Pooled libraries were sequenced on a NextSeq500 instrument (Illumina, San Diego, CA, USA) in 1 × 75 bp single‐end sequencing mode. Approximately 36 million reads were generated per sample. Reads in fastq format were aligned to the mouse reference genome version GRCm38^13^ with Gencode mV23 annotations^14^ using STAR aligner^15^ version 2.6.1a in 2‐pass mode. Reads per gene were counted by STAR, and differential gene expression was calculated using DESeq2^16^ version 1.22.2. Differently expressed genes (DEGs) were identified by q values (adjusted for multiple testing) smaller than 0.15 and a heat map with hierarchical clustering (Pearson) was created using the online‐tool Morpheus.^17^ For pathway and gene ontology analysis, DEGs were imported into the online‐tool EnrichR^18^ and regulated processes were identified by pathway enrichment using Kyoto Encyclopedia of Genes and Genomes (KEGG) 2019 as well as gene ontology analysis using the GO biological process database 2018.

### 2.9 Histological staining of murine spleen tissue

Spleens were embedded in Tissue‐Tek O.C.T. compound (Sakura Finetek, Alphen aan den Rijn, Zuid‐Holland, The Netherlands) and slowly frozen at −20°C for storage. After cutting 10 μm slides on a cryo‐microtome, the tissue samples were fixed with acetone at −20°C for 10 min and stored at room temperature afterwards. DNA fragmentation was assessed using the DeadEnd Colorimetric TUNEL system (Promega, Madison, WI, USA) following the manufacturers' manual and counterstained for 1.5 min using Mayer's hematoxylin (Morphisto, Offenbach am Main, Hessen, Germany). For immunofluorescent co‐staining of Ly6C with intracellular proteins, rabbit primary antibodies against either BIRC6 (4 μg/ml, Bethyl Laboratories, Montgomery, TX, USA) or cleaved caspase 3 (0.115 μg/ml, Cell Signaling Technology, Danvers, MA, USA) were added together with a rat antibody against Ly6C (1.66 μg/ml, Biolegend, San Diego, CA, USA) and incubated for 1 h at room temperature. Secondary donkey antibodies against rabbit IgG (2.5 μg/ml, DyLight550, Thermo Fisher Scientific, Waltham, MA, USA) and rat IgG (2.5 μg/ml, DyLight650, Thermo Fisher Scientific, Waltham, MA, USA) as well as DAPI (1 μg/ml, Sigma‐Aldrich, St. Louis, MO, USA) were added and slides were incubated for 1 h at room temperature. The slides were covered with Aqueous Mounting Medium (Abcam, Cambridge, Cambridgeshire, United Kingdom) and images were taken on a TissueFAXS automated slide scanner (TissueGnostics, Vienna, Vienna, Austria) mounted with a Zeiss ×20 objective (0.5 NA), a Baumer HXG40c brightfield and a Hamamatsu ORCA‐Flash 4.0 fluorescence light camera. Confocal images were taken on a Zeiss LSM700 confocal laser microscope mounted with a ×63 oil objective (1.4 NA) using a pinhole of 1 airy unit. The pictures were exported as 16‐bit single‐channel tiff files and were analyzed with FIJI^19^ for protein quantification on Ly6C^+^ cells.

### 2.10 Histological staining of isolated human monocytes

Isolated monocytes were seeded in RPMI1640 cell culture medium (Thermo Fisher Scientifc, Waltham, MA, USA) supplemented with 0.5% FBS (MP Biomedicals, Santa Ana, CA, USA) into Nunc LabTek 8 chamber slides (Thermo Fisher Scientific, Waltham, MA, USA) and incubated for 24 h either in the presence or absence of 100 ng/ml human recombinant Interleukin‐4 (Biolegend, San Diego, CA, USA). Afterwards, the cells were fixed and permeabilized using 4% formaldehyde/0.5% Triton‐X (both Sigma‐Aldrich, St. Louis, MO, USA). Intracellular NFκBp65 was stained using a rabbit primary antibody against p65 (10 μg/ml, Abcam, Cambridge, Cambridgeshire, United Kingdom) which was incubated for 1 h at room temperature as described previously.^20^ A secondary donkey antibody against rabbit IgG (2.5 μg/ml, DyLight550, Thermo Fisher Scientific, Waltham, MA, USA) as well as phalloidin—iFluor488 (1:1000, Abcam, Cambridge, Cambridgeshire, United Kingdom) and DAPI (1 μg/ml, Sigma‐Aldrich, St. Louis, MO, USA) were added and slides were incubated for 1 h at room temperature. The slides were covered with Aqueous Mounting Medium (Abcam, Cambridge, Cambridgeshire, United Kingdom) and confocal images were taken on a Zeiss LSM700 confocal laser microscope mounted with a ×63 oil objective (1.4 NA) using a pinhole of 1 airy unit. The pictures were exported as 16‐bit single‐channel tiff files and were analyzed with FIJI^19^ for protein quantification.

### 2.11 In vivo studies

All animals were used between 8 and 16 weeks of age. Lifespan of cells was assessed using 50 μg/g body weight of BrdU (Biolegend, San Diego, CA, USA). BrdU staining was analyzed at different time points using the Phase‐Flow Alexa Fluor 647 BrdU Kit (Biolegend, San Diego, CA, USA) as well as surface antibodies to gate for different leukocyte populations. For subcutaneous IL‐4 application, osmotic pumps (Alzet 1007D, Durect Corporation, Cupertino, CA, USA) were implanted, filled either with IL‐4 (Miltenyi Biotec, Bergisch Gladbach, Nordrhein‐Westfalen, Germany) or vehicle only. IL‐4 was applied at 2.1 μg/kg/h over 5 days to achieve a concentration of approximately 200 pg/ml. Calculations were based on pharmacokinetic studies on recombinant IL‐4 in human patients^21^ and dose translation to mouse was done using the method published by Reagan‐Shaw.^22^ For the analysis of monocyte recruitment upon a mild sterile inflammatory stimulus, an intraperitoneal injection of 0.5 ml thioglycollate was performed, utilizing a modified protocol previously described by us.^23^ Twelve hours after thioglycollate application, the peritoneal cavity was flushed with PBS supplemented with 3 mM EDTA and cells were analyzed by flow cytometry.

### 2.12 Human patient cohort

Monocyte counts were measured from venous blood samples using an XE‐5000 hematology analyzer (Sysmex, Vienna, Vienna, Austria). IL‐4 levels were measured previously in the Vienna CATS study,^24^ using a Human IL‐4 Magnetic Luminex Assay (R&D Systems, Minneapolis, MN, USA) on a Luminex Analyzer.

### 2.13 Chemotaxis Boyden chamber assay

Migration upon a chemotactic stimulus was assessed using the QCM Chemotaxis Cell Migration Assay with 5 μm pore size (Merck, Kenilworth, NJ, USA) following the manufacturers' protocol with 4‐h incubation at 37°C. The following conditions were studied: cell culture medium (RPMI 1640 + 1% BSA) alone, cell culture medium supplemented with 100 ng/ml IL‐4, and cell culture medium supplemented with 10% FBS. Total cell amounts were quantified by flow cytometry of a defined volume using an Attune NxT flow cytometer.

### 2.14 Quantitative PCR

RNA from cell culture lysates was prepared using Maxwell RSC simplyRNA Tissue kits on a Maxwell RSC Instrument (both Promega, Madison, WI, USA). cDNA was created using a 1:1 Oligo(dT)/Random Primer Reverse Transcriptase Mix (Promega, Madison, WI, USA). qPCR was performed on a CFX Connect Real‐Time PCR Detection System (Bio‐Rad Laboratories, Hercules, CA, USA). For the detection of different genes, primers were designed using Universal Probe Library (UPL) system (Roche, Basel, Switzerland) and qPCR was performed using GoTaq Probe qPCR Master Mix (Promega, Madison, WI, USA). Primer sequences and used UPL probes were as following: human/murine 18S (FWD‐gggttcgattccggagag, REV‐tcgggagtgggtaatttgc, UPL 40), human GAPDH (FWD‐agccacatcgctcagacac, REV‐gcccaatacgaccaaatcc, UPL 60), human Beta‐actin (FWD‐ccaaccgcgagaagatga, REV‐ccagaggcgtacagggatag, UPL 64), murine GAPDH (FWD‐agcttgtcatcaacgggaag, REV‐tttgatgttagtggggtctcg, UPL 9), and murine Beta‐actin (FWD‐ctaaggccaaccgtgaaaag, REV‐accagaggcatacagggaca, UPL 64), murine IL‐1α (FWD‐ttggttaaatgacctgcaaca, REV‐gagcgctcacgaacagttg, UPL 52), murine MCP‐1 (FWD‐catccacgtgttggctca, REV‐gatcatcttgctggtgaatgagt, UPL 62), human IL‐1α (FWD‐ggttgagtttaagccaatcca, REV‐tgctgacctaggcttgatga, UPL 6), human MCP‐1 (FWD‐ttctgtgcctgctgctcat, REV‐ggggcattgattgcatct, UPL 83), murine BIRC6 (FWD‐ ctttgcagtctctctctcatgc, REV‐ gcttccagctttgctttctg, UPL 41). *C*_*t*_ values were calculated by regression and normalized by the ΔΔC_*t*_ method onto 18S, GAPDH and Beta‐actin as housekeeping genes and relativized to unstimulated monocytes.

### 2.15 Statistics

Statistical analysis was performed using GraphPad Prism 8. Normality was assessed using D'Agostino–Pearson test (*n* ≥ 8) or Shapiro–Wilk test (*n* < 8). For normally distributed data an unpaired or paired *t*‐test was calculated comparing two groups, or one‐way ANOVA was performed for more than two groups. Non‐normally distributed data were assessed with Mann–Whitney test for two groups. Data on antibody arrays (cytokines, apoptosis) were corrected for multiple testing using the two‐stage linear step‐up procedure of Benjamini, Krieger, and Yekutieli. For comparison of the genotypes at different time points in the kinetic studies, a two‐way ANOVA was performed with Tukey correction for multiple testing. For Pearson's correlation coefficient a linear regression was calculated in GraphPad Prism 8. Unless otherwise stated, summary data are always shown as mean ± SD.

## 3 RESULTS

### 3.1 Circulating monocyte numbers are reduced in IL‐4Rα knockout mice

**FIGURE 1:**
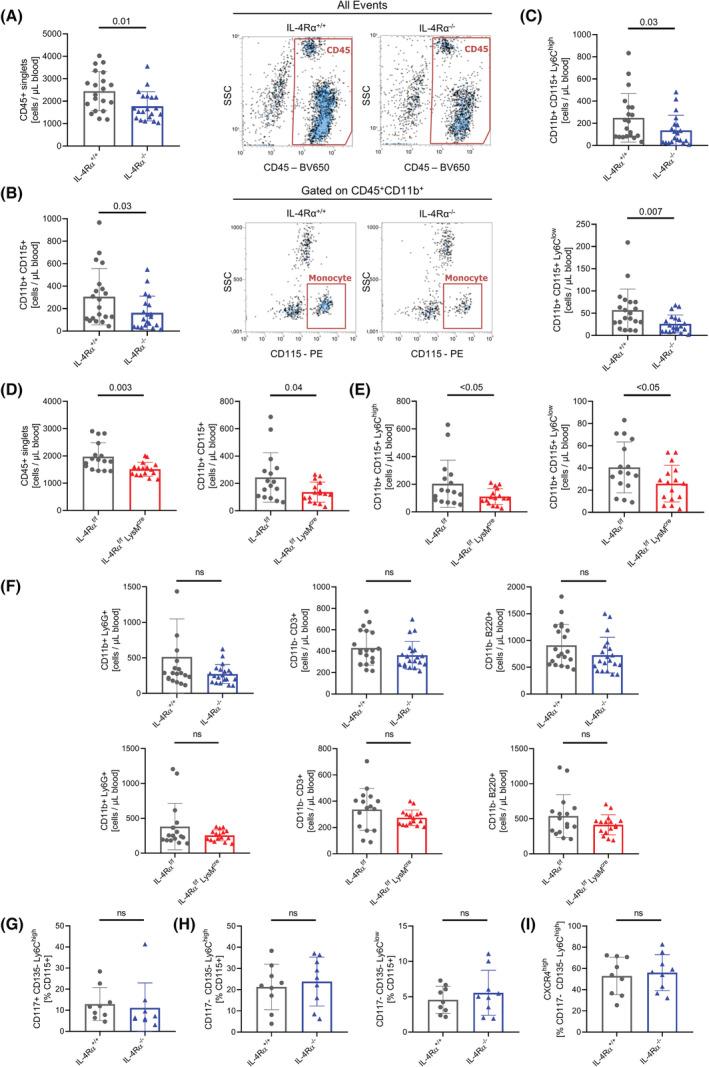
Circulating monocyte numbers are reduced in IL‐4Rα knockout mice. (A–C) Flow cytometry analysis of absolute numbers of CD45^+^ leukocytes (A), CD11b^+^CD115^+^ monocytes (B), and monocyte subsets (C) in the blood of IL‐4Rα^+/+^ and IL‐4Rα^−/−^ mice (*n* = 20 per genotype). (D,E) Flow cytometry analysis of absolute numbers of CD45^+^ leukocytes and CD115^+^ monocytes (D) and monocyte subsets (E) in the blood of IL‐4Rα^f/f^ and IL‐4Rα^f/f^ LysM^cre^ mice (*n* = 16 per genotype). (F) Flow cytometry analysis of absolute numbers of CD11b^+^Ly6G^+^ granulocytes, CD11b^−^CD3^+^ T cells and CD11b^−^B220^+^ B cells in the blood of IL‐4Rα^+/+^ and IL‐4Rα^−/−^ mice (*n* = 20 per genotype) as well as in the blood of IL‐4Rα^f/f^ and IL‐4Rα^f/f^ LysM^cre^ mice (*n* = 16 per genotype). (G–I) Flow cytometry analysis of relative amount of CD115^+^CD117^+^CD135^−^Ly6C^high^ common monocyte precursor cells (G), CD115^+^CD117^−^CD135^−^ monocyte subsets (H), and CD115^+^Ly6C^high^CXCR4^high^ monocytes (I) in the bone marrow of IL‐4Rα^+/+^ and IL‐4Rα^−/−^ mice (*n* = 9 per genotype, ns = not significant).

Leukocyte populations in the blood of two different IL‐4Rα knockout mouse lines^9^, ^10^ were analyzed (Figure S1A and for gating S1B). Compared to WT, IL‐4Rα^−/−^ mice had a significant reduction of circulating CD45^+^ leukocytes (Figure 1A). From the CD45+ cell populations analyzed, CD11b^+^CD115^+^ monocytes were significantly reduced by 47% (Figure 1B). This reduction was observed in both the Ly6C^high^ and the Ly6C^low^ monocyte subsets (Figure 1C). Similar reductions in leukocyte populations were found in IL‐4Rα^f/f^ LysM^cre^ knockout animals (Figure 1D,E). The absolute numbers of CD11b^+^Ly6G^+^ neutrophils, CD11b^−^CD3^+^ T cells and CD11b^−^B220^+^ B cells did not differ significantly in our analysis, although on average, there was a trend toward reduction in knockout mice compared to the respective wild types (Figures 1F and S1C).

As monocytes arise from myeloid progenitor cells in the bone marrow,^25^ we assessed the bone marrow of the mice (gating Figure S1D). Adult mice lacking IL‐4Rα showed similar amounts of CD115^+^CD117^+^CD135^−^ myeloid progenitor and CD115^+^CD117^−^CD135^−^ Ly6C^high^ and Ly6C^low^ monocytes in their bone marrow, compared to respective WT littermates (Figures 1G,H and S1E). As CXC‐motif chemokine receptor 4 (CXCR4) signaling leads to the retention of (pre‐)monocytes in the bone marrow,^26^ we quantified the amount of CXCR4^high^Ly6C^high^ monocytes, but did not observe any significant differences (Figure 1I). These findings suggest that IL‐4Rα expression is related to circulating monocyte counts, but the amount of monocytes and their direct precursors (CD115^+^CD117^+^CD135^−^ cells) in bone marrow is similar between WT and IL‐4Rα knockout animals in our experiments.

### 3.2 Inflammatory cytokine levels are reduced in the plasma of IL‐4Rα‐deficient mice

**FIGURE 2:**
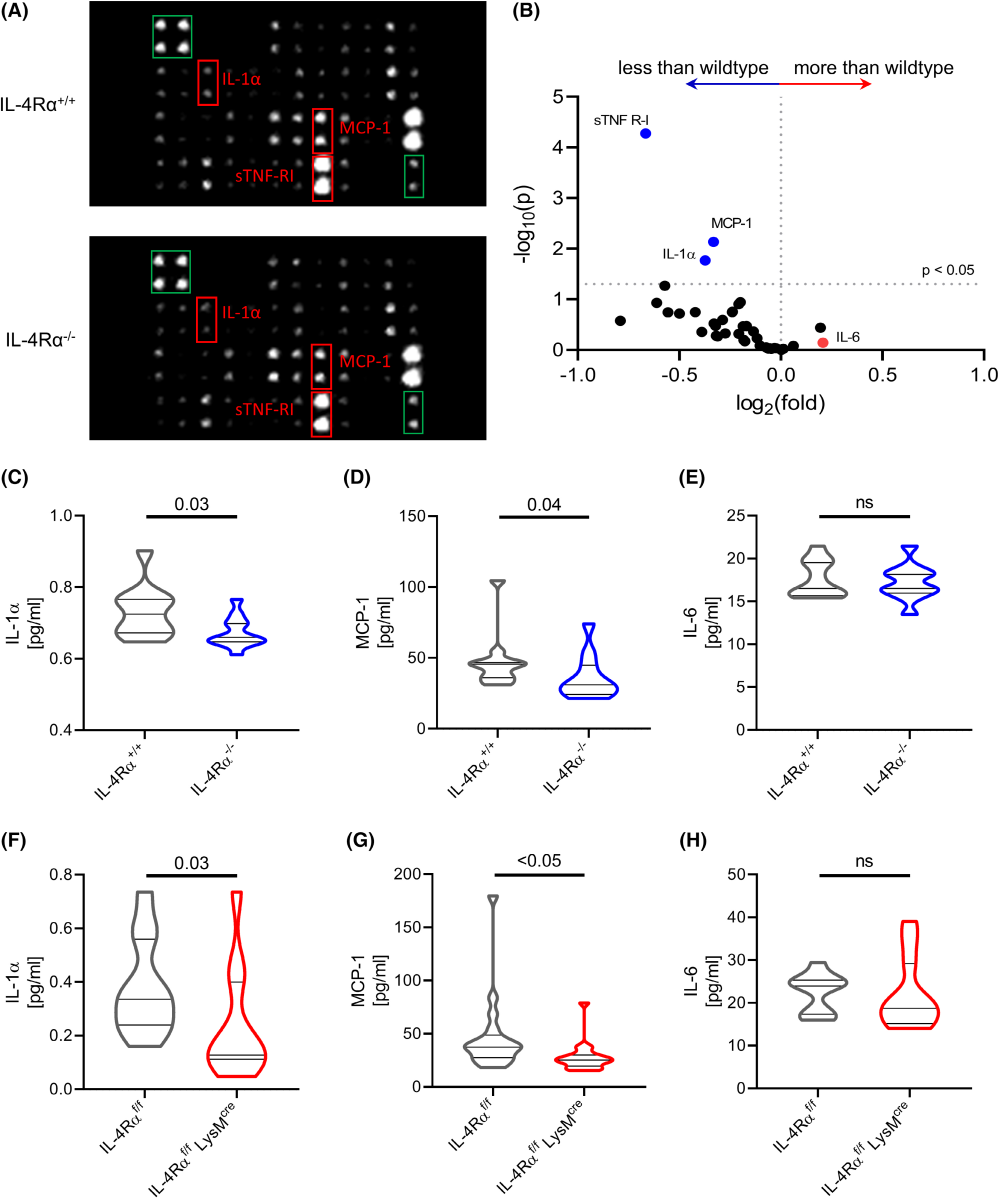
Inflammatory cytokine levels are reduced in the plasma of IL‐4Rα‐deficient mice. (A,B) Arrays of antibodies against 40 plasmatic cytokines were incubated with plasma from IL‐4Rα^+/+^ and IL‐4Rα^−/−^ mice. Red boxes mark significantly regulated cytokines, green boxes mark normalization controls (A, both images were adjusted for brightness and contrast equally). Volcano plot of the cytokine array, cytokines of interest are labeled and marked with colors (B). (*n* = 4 per genotype) (C–E) Violin plots of IL‐1α (C), MCP‐1 (D) and IL‐6 (E) quantified in plasma of IL‐4Rα^+/+^ and IL‐4Rα^−/−^ mice (*n* = 11 per genotype, ns = not significant). (F–H) Violin plots of IL‐1α (F), MCP‐1 (G), and IL‐6 (H) quantified in plasma of IL‐4Rα^f/f^ and IL‐4Rα^f/f^ LysM^cre^ mice (*n* = 18 per genotype, ns = not significant).

We next asked, if circulating cytokine levels would be altered in IL‐4Rα knockout animals. Using a cytokine array assay compared to WT, IL‐4Rα^−/−^ mice had significantly reduced levels of inflammatory cytokines IL‐1α, monocyte chemoattractant protein‐1 (MCP‐1) and soluble tumor necrosis factor receptor‐I (sTNF‐RI) in their plasma, after correction for multiple testing (Figure 2A,B; Table S1). When quantifying IL‐1α and MCP‐1 plasma levels by ELISA (Figure 2C,D) we could confirm the significant downregulation of these cytokines in IL‐4Rα^−/−^ mice that was seen when using the cytokine array. We also quantified IL‐6 levels by ELISA, as this cytokine was the most upregulated hit in the array data, but similar to the cytokine array, IL‐6 levels did not significantly differ between WT and IL‐4Rα^−/−^ mice (Figure 2E). To evaluate the specific, myeloid cell‐intrinsic contribution for altered cytokine levels, we quantified IL‐1α, MCP‐1, and IL‐6 in IL‐4Rα^f/f^ LysM^cre^ mice. In line with the full body knockout, IL‐1α (Figure 2F) and MCP‐1 (Figure 2G) levels were reduced in the plasma of IL‐4Rα^f/f^ LysM^cre^ mice, whereas we did not observe any difference in IL‐6 levels (Figure 2H). sTNF‐RI was only significantly reduced in IL‐4Rα^−/−^ but not in IL‐4Rα^f/f^ LysM^cre^ mice (Figure S1F).

To assess if IL‐4 directly influences the expression of IL‐1α, IL‐6, or MCP‐1 by monocytes, we stimulated human (Figure S2A) as well as murine (Figure S2B) monocytes with IL‐4 and quantified the transcription of IL‐1α (*IL1A*), IL‐6 (*IL6*) as well as MCP‐1 (*CCL2*), but no significant induction in mRNA expression by IL‐4 was observed. Summarizing, we found that both our IL‐4Rα deficient mouse strains had reduced amounts of MCP‐1 and IL‐1α in their plasma.

### 3.3 IL‐4Rα knockout alters transcriptome of splenic monocytes

**FIGURE 3:**
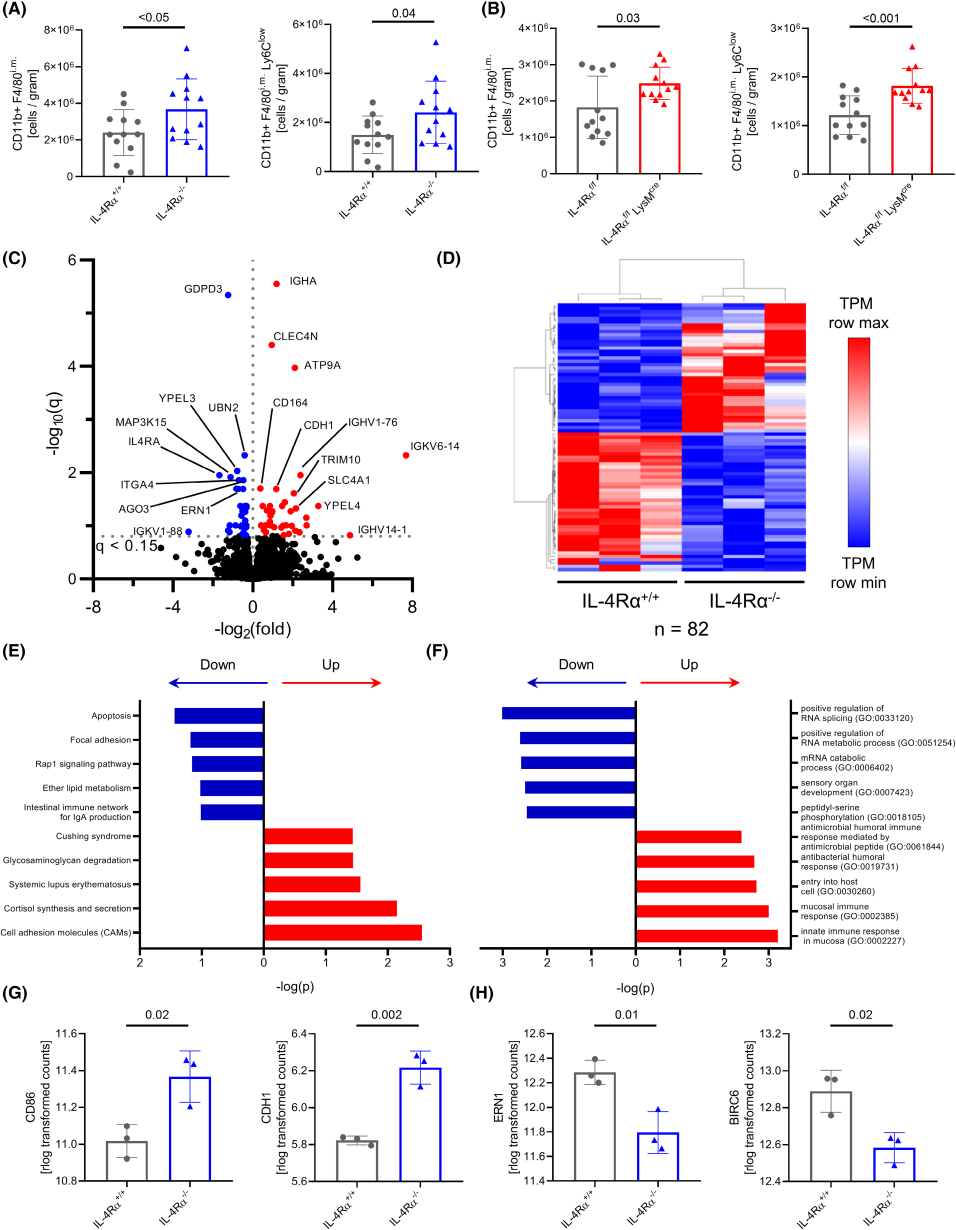
IL‐4Rα knockout alters transcriptome of splenic monocytes. (A,B) Flow cytometry analysis of absolute numbers of CD11b^+^F4/80^i.m^ and CD11b^+^F4/80^i.m^Ly6C^low^ monocytes in the spleens of IL‐4Rα^+/+^ and IL‐4Rα^−/−^ mice (A, *n* = 12 per genotype) and IL‐4Rα^f/f^ and IL‐4Rα^f/f^ LysM^cre^ mice (B, *n* = 12 per genotype). (C–H) RNAseq analysis of splenic monocytes from IL‐4Rα^+/+^ and IL‐4Rα^−/−^ mice. Volcano plot of differently regulated genes. Color dots represent DEGs (C). Pearson's hierarchical clustering (D). Top significant altered KEGG pathways (E). Top significant altered GO terms (F). Counts (rlog transformed) per gene for targets *CD86* and *CDH1* identified as DEGs in the KEGG “Cell adhesion molecules (CAMs)” pathway (G). Counts (rlog transformed) per gene for the targets *ERN1* and *BIRC6* identified as DEGs in the KEGG “Apoptosis” pathway (H) (C–H, *n* = 3 per genotype).

In addition to the circulation, monocytes are also present in a splenic reservoir during homeostasis.^27^, ^28^ Therefore, we analyzed the monocyte content in spleens of both IL‐4Rα knockout lines (gating Figure S2C). Increased numbers of splenic monocytes, defined as CD11b^+^F4/80^intermediate^ cells, were found in IL‐4Rα^−/−^ and IL‐4R^f/f^ LysM^cre^ mice, compared to respective WT littermates. By further dividing of the CD11b^+^F4/80^intermediate^ monocytes by their Ly6C expression, we found that only the Ly6C^low^ monocyte subset was significantly increased in IL‐4Rα^−/−^ and IL‐4R^f/f^ LysM^cre^ mice (Figure 3A,B), whereas no significant differences were observed for Ly6C^high^ monocytes (Figure S2D).

To identify transcriptional changes in the absence of IL‐4Rα, splenic monocytes were analyzed by bulk RNA sequencing. The overall transcriptome of IL‐4Rα^−/−^ splenic monocytes was not significantly altered compared to WT, as no clustering between samples was observed in principle component analysis (Figure S2E). We identified 82 differently expressed genes (DEGs, Figure 3C,D) using DESeq2,^16^ which were then further analyzed using the EnrichR tool.^18^ The most significantly altered pathways in KEGG analysis were connected to “Apoptosis” as well as “Cell adhesion molecules (CAMs)” (Figure 3E). Furthermore, GO analysis showed an overrepresentation of antibacterial gene sets compatible with a shift toward T_helper_‐1 responses (Figure 3F). Analysis of the individual genes related to the KEGG pathway “Cell adhesion molecules (CAMs)” showed an increased expression of *CD86* (or B7.2) as well as *CDH1* (or E‐cadherin) in IL‐4Rα‐deficient monocytes (Figure 3G).

As KEGG pathway analysis revealed changes in apoptosis pathways, we determined the DEGs involved in the “Apoptosis” KEGG pathway in our dataset. Two anti‐apoptotic genes, *ERN1* as well as *BIRC6*, were significantly downregulated in monocytes lacking IL‐4Rα (Figure 3H). Collectively, these findings showed that lack of IL‐4Rα impacts splenic monocyte amounts. Furthermore, transcriptional analysis of isolated monocytes from spleens suggested alterations in the apoptosis pathway due to the lack of IL‐4Rα.

### 3.4 Increased cell death of splenic monocytes in the absence of IL‐4Rα signaling

**FIGURE 4:**
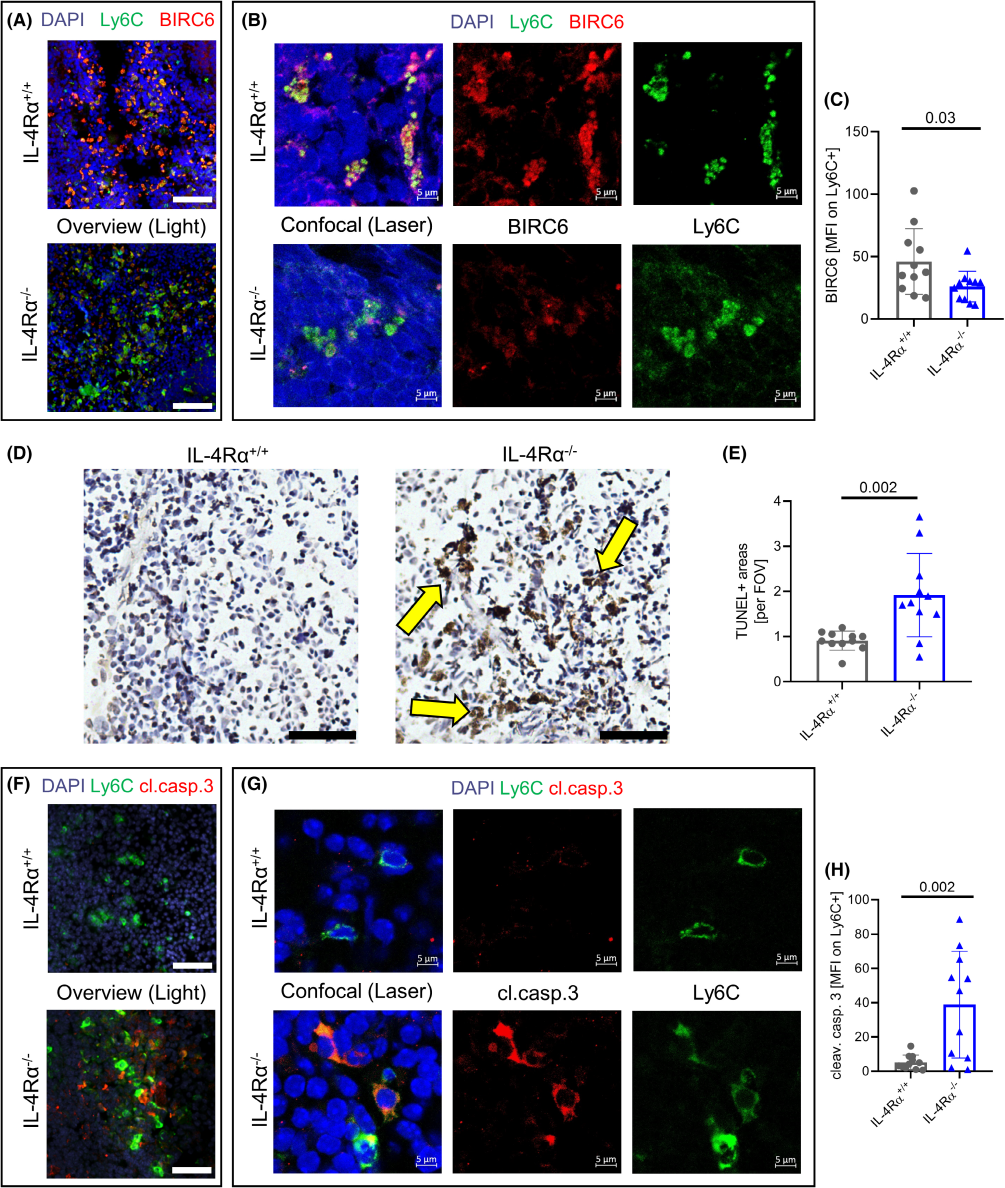
Increased cell death of splenic monocytes in the absence of IL‐4Rα signaling. (A–C) Immunofluorescent co‐staining of Ly6C and BIRC6 in the spleen of IL‐4Rα^+/+^ and IL‐4Rα^−/−^ mice acquired on a TissueFAXS Zeiss observer light microscope; scale bars = 50 μm (A). Confocal laser microscopy of the same samples as in A showing colocalization of BIRC6 with Ly6C signal at a higher magnification acquired on a LSM700 Zeiss laser microscope; scale bars = 5 μm (B). The mean fluorescent intensity of BIRC6 at areas positive for Ly6C (C). (D,E) Immunohistochemical TUNEL staining in the spleen of IL‐4Rα^+/+^ and IL‐4Rα^−/−^ mice acquired on a TissueFAXS Zeiss observer light microscope; scale bars = 50 μm. Yellow arrows indicate TUNEL^+^ areas (D). TUNEL^+^ areas were blindly analyzed in 20 fields of view per sample (E). (F–H) Immunofluorescent co‐staining of Ly6C and cleaved caspase 3 in the spleen of IL‐4Rα^+/+^ and IL‐4Rα^−/−^ mice acquired on a TissueFAXS Zeiss observer light microscope; scale bars = 50 μm (F). Confocal laser microscopy of the same samples as in F showing colocalization of cleaved caspase 3 with Ly6C signal at a higher magnification acquired on a LSM700 Zeiss laser microscope; scale bars = 5 μm (G). The mean fluorescent intensity of cleaved caspase 3 at areas positive for Ly6C was analyzed (H) (*n* = 11 per genotype).

As our RNAseq data of splenic monocytes suggested alterations in apoptosis, we aimed to confirm the findings by quantification of apoptosis‐related transcripts in the spleen. IL‐4Rα‐deficient monocytes indeed expressed lower amounts of the anti‐apoptotic protein BIRC6, identified as significantly downregulated DEG in the RNAseq analysis (Figure 4A–C). Furthermore, DNA fragmentation, which is typically generated during apoptosis, was significantly increased in IL‐4Rα^−/−^ mice, compared to WT littermates (Figure 4D,E). Cleavage of caspase‐3 is a central mechanism in programmed cell death.^29^ Cleaved caspase‐3 was strikingly increased in areas staining positive for Ly6C in spleens of IL‐4Rα^−/−^ mice compared to WT littermates (Figure 4F–H), which was verified also by flow cytometric analysis of monocytes in splenic single cell suspensions (Figure S2F). Thus, monocytes in the spleen of IL‐4Rα deficient mice showed increased markers of cell death also on protein level, compared to wild‐type littermates.

### 3.5 IL‐4 prevents apoptosis of human monocytes

**FIGURE 5:**
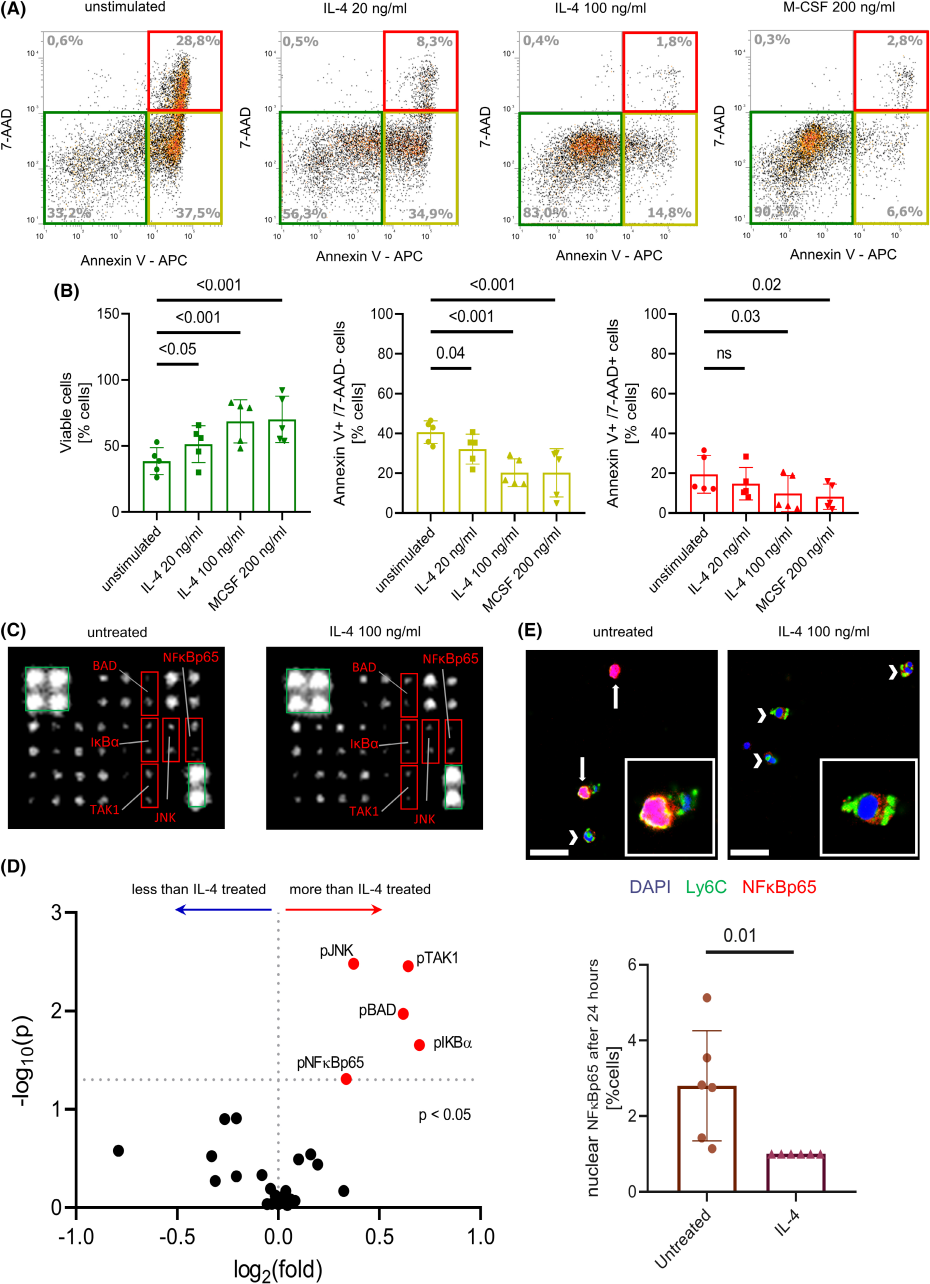
IL‐4 prevents apoptosis of human monocytes. (A,B) AnnexinV/7‐AAD staining of human monocytes. The colored boxes indicate viable (green), early apoptotic/necrotic (yellow), and late apoptotic/necrotic (red) cells (A). Flow cytometry analysis of the AnnexinV/7‐AAD staining (B). (*n* = 5 human donors, ns = not significant) (C,D) 19 signaling proteins involved in the apoptosis pathway were analyzed in lysates of human monocytes treated with 100 ng/ml IL‐4 for 24 h. Red boxes mark significantly regulated proteins, green boxes mark normalization controls (C, both images were adjusted for brightness and contrast equally). Volcano plot of the protein array, proteins of interest are labeled and marked with colors (D). (*n* = 4 human donors) (E) Confocal laser microscopy of the immunofluorescence staining and quantification of NFκBp65 in isolated human monocytes either left untreated or treated with 100 ng/ml IL‐4 for 24 h; scale bars = 20 μm. A 6× higher magnification of some cells is shown in the canvas for each condition. (*n* = 6 human donors).

To investigate whether IL‐4 directly affects the survival of monocytes and if the observed findings are conserved between species, we stimulated human monocytes in vitro using IL‐4. IL‐4 treatment significantly increased the proportion of viable cells, being comparable to the stimulation of monocytes using M‐CSF (Figure 5A,B). To identify potentially signaling pathways affected by IL‐4, the phosphorylation of proteins involved in apoptosis was assessed. Compared to IL‐4 stimulated monocytes, significantly more phosphorylated JNK, BAD, TAK1, NFκBp65, and IκBα was detected in unstimulated cells after correction for multiple testing (Figure 5C,D; Table S2). To assess if the different phosphorylation of these targets would also lead to a change in the activation state of the NF‐κB/IκB pathway in monocytes treated with IL‐4, nuclear p65 was quantified by confocal microscopy in monocytes which were either left untreated or were treated for 24 h with IL‐4. Unstimulated monocytes showed significantly increased nuclear p65 signal after 24 h compared to IL‐4 treated cells (Figure 5E), which is in line with reports showing that phosphorylation of IκBα and NFκBp65 facilitates the transport of p65 into the nucleus.^30^ Taken together, these data suggest that IL‐4 promotes the survival of human monocytes in vitro and that stimulation with IL‐4 for 24 h leads to reduced phosphorylation of pro‐apoptotic intracellular pathways in these cells compared to cells left unstimulated.

### 3.6 IL‐4Rα signaling in monocytes directly regulates their lifespan in vivo

**FIGURE 6:**
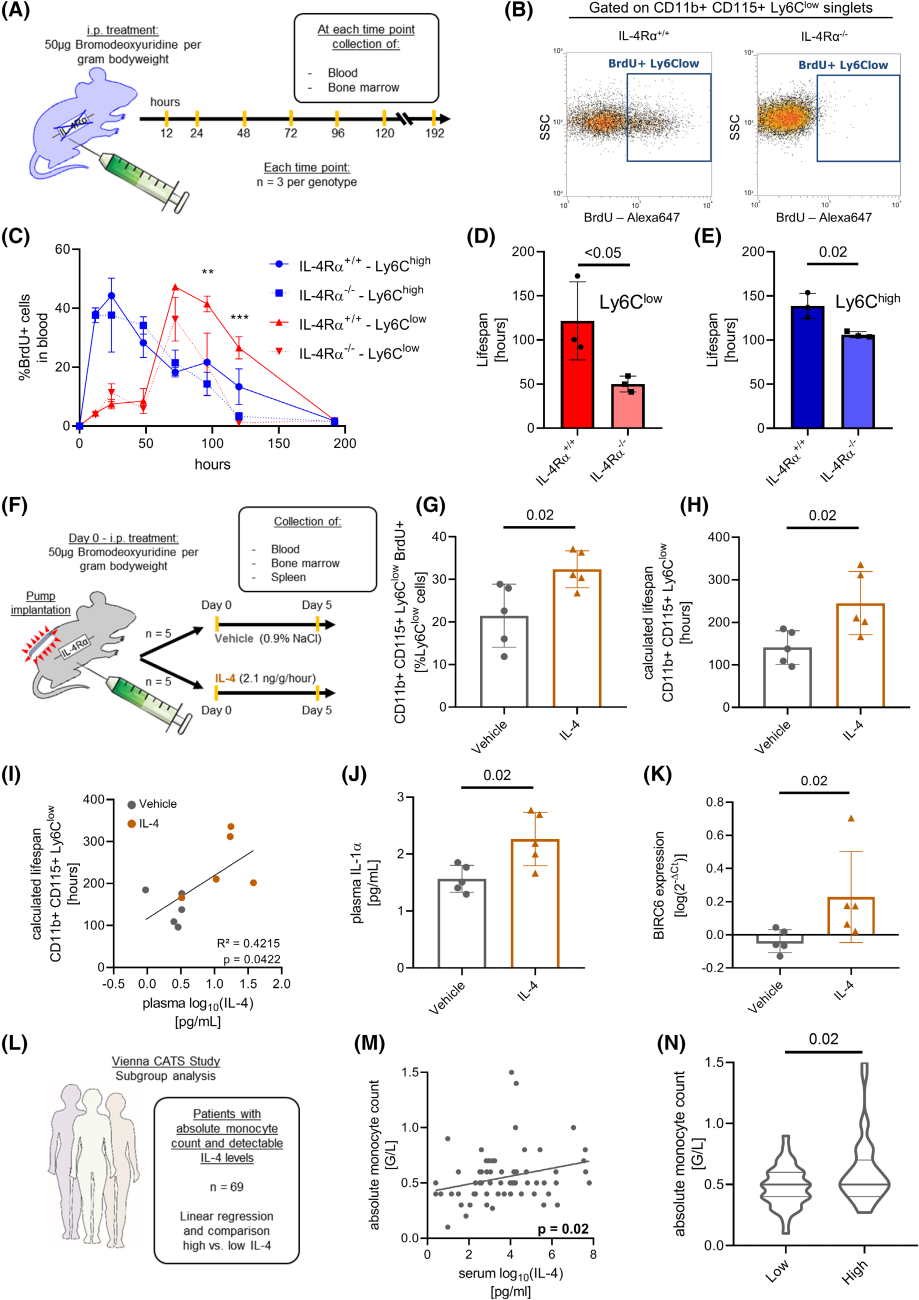
IL‐4Rα signaling in monocytes directly regulates their lifespan in vivo. (A–E) Experimental setup of lifespan measurement (A). Flow cytometry plot of BrdU signal 120 h after BrdU injection (B). Time kinetic of BrdU means ± SEM in monocytes in blood. Asterisks indicate significant differences in Ly6C^low^ subset between IL‐4Rα^+/+^ and IL‐4Rα^−/−^, calculated by two‐way ANOVA (C, **p < .01, ***p < .001). Calculated lifespan of the Ly6C^low^ (D) and Ly6C^high^ (E) monocyte subsets. (*n* = 3 per genotype and time point) IL‐4 application regulates lifespan of monocytes in vivo (F–K) Experimental setup of IL‐4 application (F). Flow cytometry analysis of BrdU signal in CD11b^+^CD115^+^Ly6C^low^ monocytes (G). Lifespan of Ly6C^low^ monocytes (H). Linear regression of plasma IL‐4 levels and lifespan of CD11b^+^CD115^+^Ly6C^low^ monocytes (I). IL‐1α in plasma (J). *BIRC6* expression in the spleen (K) (*n* = 5 per treatment). (L–N) Association of serum IL‐4 levels with absolute monocyte counts in humans (L). Linear regression of log_10_ (IL‐4) levels in serum with absolute monocyte counts (M). Violin plot of monocyte counts stratified by IL‐4 (*n* = 69 patients) (N).

We next asked if the lifespan of circulating monocytes in IL‐4Rα^−/−^ mice is altered in vivo. Therefore, bromodeoxyuridine (BrdU) was administered to determine the kinetics of leukocyte subpopulations (Figure 6A). One hundred and twenty hours after BrdU injection a higher proportion of Ly6C^low^ monocytes in WT mice was still labeled with BrdU, compared to IL‐4Rα^−/−^ animals (Figure 6B). In general, the amount of BrdU^+^ Ly6C^low^ monocytes decreased significantly faster in IL‐4Rα‐deficient mice (Figure 6C). The lifespan of Ly6C^low^ monocytes at baseline in IL‐4Rα^−/−^ mice was reduced by 55%, compared to IL‐4Rα^+/+^ mice (Figures 6D and S3A). Similarly, Ly6C^high^ monocytes of IL‐4Rα‐deficient mice showed a 21% reduction in estimated lifespan (Figure 6E). The lifespan of granulocytes and lymphocytes in the blood was not significantly affected (Figure S3B) and no differences were found in the bone marrow (Figure S3C,D).

We next examined if subcutaneous application of IL‐4 would prolong the lifespan of blood monocytes (Figure 6F). Subcutaneous IL‐4 treatment led to a steep increase in plasmatic IL‐4 levels (Figure S3E). In the IL‐4 treated group, a significantly greater amount of Ly6C^low^ monocytes was still labeled with BrdU, compared to the vehicle group (Figure 6G). IL‐4 treatment increased the lifespan of Ly6C^low^ monocytes to 174%, compared to vehicle‐treated animals (Figure 6H) and the calculated lifespan of Ly6C^low^ monocytes significantly correlated (*R* ^2^ = 0.4215) with the amount of IL‐4 in plasma (Figure 6I). Additionally, we measured increased levels of IL‐1α in plasma of mice treated with IL‐4 (Figure 6J). *BIRC6* mRNA expression in the spleen was significantly increased in mice treated with IL‐4 compared to the vehicle group (Figure 6K). When analyzing the BrdU content of bone marrow cells, we again did not observe any significant differences between IL‐4 or vehicle‐treated mice (Figure S3F). Of note, subcutaneous application of IL‐4 into mice prolonged the lifespan of Ly6C^low^ monocytes in WT mice. To summarize, the lifespan of monocytes together with the number of total circulating monocytes was reduced in mice lacking IL‐4Rα.

### 3.7 Monocyte numbers are associated with IL‐4 serum levels in humans

We evaluated a possible association of IL‐4 levels in serum with absolute blood monocyte counts in the study cohort of the Vienna Cancer and Thrombosis Study (CATS).^11^ Patients with available data on monocyte counts and IL‐4 levels at study baseline were included in this exploration, resulting in an analysis cohort of 458 patients (Figure S3G). Characteristics of the analysis cohort are provided in Table S3, and the distribution of monocyte counts and IL‐4 levels obtained at study baseline are shown in Figure S3H. We stratified the analysis cohort into patients with high IL‐4 levels (top 10%) and patients with low/undetectable IL‐4 levels (other 90%) for the comparison of monocyte counts in the total analysis cohort (*n* = 458). Patients with high levels of IL‐4 had significantly elevated blood levels of monocytes compared to patients with low IL‐4 levels (0.6 G/L [95%CI: 0.5–0.7] vs. 0.5 G/L [95%CI: 0.5–0.5], *p* = .044) (Figure S3I). We further studied the subgroup of patients with detectable IL‐4 levels (*n* = 69, 15% of the total analysis cohort) in detail (Figure 6L). A significant association between monocyte counts and IL‐4 levels was observed, with an increase in monocytes by 0.04 G/L (95%CI: 0.01–0.06, *p* = .023) per log_10_ increase in IL‐4 levels in this subgroup (Figure 6M). By stratifying the subgroup comparing patients with IL‐4 levels above the median (>21.87 pg/ml) to patients with levels at or below the median, we found that monocyte counts were significantly higher in patients with IL‐4 levels above the cut‐off (Figure 6N). Importantly, these two groups did not differ significantly in terms of age, sex, tumor type, diagnostic setting or the presence of metastatic disease (Table S4).

### 3.8 Lack of IL‐4Rα in myeloid cells leads to impaired recruitment of monocytes into the peritoneal cavity

**FIGURE 7:**
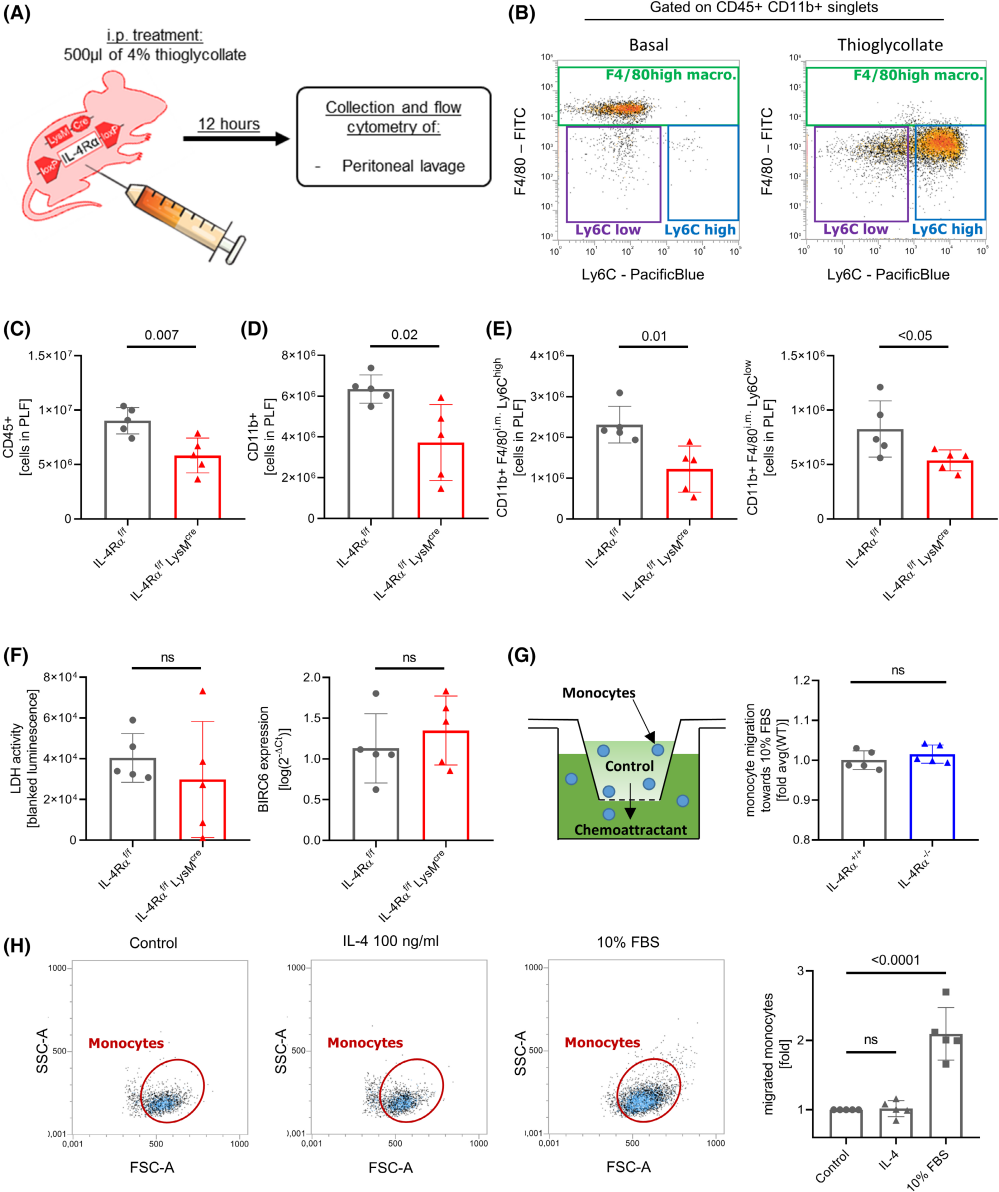
Lack of IL‐4Rα in myeloid cells leads to impaired recruitment of monocytes into the peritoneal cavity. (A) Experimental setup. (B) Representative flow cytometry plot of the peritoneal lavage fluids. (C–E) Flow cytometry analysis of absolute numbers of CD45^+^ leukocytes (C), CD11b^+^ myeloid cells (D), and monocyte subsets (E) in the peritoneal lavage fluid 12 h after thioglycollate injection. (*n* = 5 per group) (F) Assessment of cell death parameters in the peritoneal lavage fluid (PLF) of the thioglycollate experiment. Lactate dehydrogenase activity was measured in the supernatant of the PLF after centrifugation by measuring the luminescence signal of a specific substrate on a Promega GloMax reader (left). *BIRC6* gene expression was analyzed by qPCR in the lysate of the cell suspension of the PLF after centrifugation (right). (*n* = 5 per group) (G) Experimental setup of a Boyden chamber assay and assessment of the overall migratory capacity of murine monocytes from IL‐4Rα^−/−^ mice and WT littermates against 10% FBS in vitro using a commercial Boyden chamber assay. (*n* = 5 per group) (H) Representative flow cytometry plots and analysis of flow cytometry data of migration experiments using human isolated monocytes (*n* = 5 individual human donors).

Given the role of IL‐4Rα in the survival and regulation of circulating monocyte numbers in vivo, we next assessed if the observed decrease in monocytes impacts on their recruitment in a sterile peritonitis model (Figure 7A). The myeloid‐restricted IL‐4Rα knockout line was used to rule out additional effects of IL‐4 on the extravasation of leukocytes, for example by increasing endothelial permeability.^31^

Twelve hours after thioglycollate injection, Ly6C^high^ as well as Ly6C^low^ monocytes were recruited in significant numbers to the peritoneal cavity (Figure 7B). Compared to WT littermates, IL‐4Rα^f/f^ LysM^cre^ mice recruited 35% less CD45^+^ leukocytes over 12 h (Figure 7C). This reduction was due to a significantly impaired recruitment of CD11b^+^ leukocyte populations by 41% (Figure 7D), whereas no difference was observed for lymphoid CD11b^−^ cells (Figure S4A). In the CD11b^+^ cell population, Ly6C^high^ as well as Ly6C^low^ monocytes were significantly reduced by 47% and 35%, respectively (Figure 7E), whereas no significant difference was found in CD11b^+^Ly6G^+^ granulocytes (Figure S4B) or CD11b^+^F4/80^high^ macrophages (Figure S4C). To rule out that increased cell death would be responsible for the reduced amount of monocytes in peritoneal lavage fluid (PLF), we assessed lactate dehydrogenase activity as well as *BIRC6* expression in the lysate of the recovered PLF cells. No significant difference was observed between IL‐4Rα^f/f^ LysM^cre^ mice and their wild‐type littermates (Figure 7F).

We asked if the lack of IL‐4Rα would impact on the migratory capacity of monocytes compared to wild‐type littermates. Therefore, we examined the overall migratory capacity of murine monocytes from IL‐4Rα^−/−^ mice and WT littermates against 10% FBS in vitro using a Boyden chamber assay, but did not observe a significant difference between WT and knockout animals (Figure 7G). To evaluate if local IL‐4 would act as a chemotactic factor, influencing the recruitment of monocytes in our model, we studied migration of human IL‐4R competent monocytes toward IL‐4 and a positive control (10% FBS), but there was no significant difference in transmigrating cells between the IL‐4 group and the medium control (Figure 7H), ruling out a direct chemotactic effect of IL‐4 on monocytes.

Taken together, these results suggest, that less monocytes are found in the peritoneal cavity of IL‐4Rα mice in the thioglycollate model, without significant differences in markers of cell death in the immigrated cells or in the PLF. Furthermore, IL‐4 did not act as chemotactic factor on human monocytes, nor was the overall migratory capacity of monocytes lacking IL‐4Rα reduced in our Boyden chamber experiments.

## 4 DISCUSSION

While IL‐4 and IL‐13 are known to be involved in the T_helper_‐2 response in lymphocytes and also in the polarization of macrophages into an alternatively activated state,^32^, ^33^ we identified IL‐4Rα as an important receptor for monocyte lifespan. Lack of IL‐4R signaling by deletion of IL‐4Rα reduced the amount of circulating monocytes in vivo, accompanied by a reduction in plasmatic monocyte‐derived inflammatory cytokines. Furthermore, IL‐4Rα‐deficient monocytes accumulated in the spleen of mice, accompanied by increased monocyte death. IL‐4Rα‐deficient monocytes showed a diminished lifespan, whereas application of IL‐4 to WT mice increased the lifespan of these cells in vivo, suggesting that IL‐4 signaling regulates monocyte survival. It would be of great interest, to also study monocyte lifespan in IL‐4 deficient mice, as IL‐4 and IL‐13 are both involved in the activation of IL‐4R. Stimulation of human monocytes with IL‐4 prevented the induction of apoptosis in vitro. In a human clinical cohort, serum IL‐4 levels were significantly associated with monocyte counts in blood, supporting the role of IL‐4 receptor signaling in monocyte survival observed in our mouse experiments. Reduced circulating monocyte counts in IL‐4Rα‐deficient mice had also an impact on monocyte recruitment, as the accumulation of monocytes in the peritoneal cavity upon thioglycollate application was diminished. Thus, we describe an unexpected function of IL‐4Rα on monocytes during homeostasis, which could potentially be used to identify new therapeutic approaches in diseases with monocyte contribution.

Regulation of circulating monocyte numbers in vivo depends on a counterbalance of basal as well as chemokine‐stimulated generation and release of new cells from the bone marrow on the one hand and extravasation of monocytes or death of the cells on the other.^34^ We identified a regulatory mechanism involved in the maintenance of monocyte counts by showing that the amount of circulating monocytes is reduced in the absence of IL‐4Rα on these cells and that this reduction is caused by a diminished lifespan of IL‐4Rα‐deficient monocytes. Under steady state conditions, the amount of IL‐4 in the circulation is very low, compared to conditions such as parasite infections.^35^ However, knockout of IL‐4Rα reduced the lifespan of Ly6C^low^ monocytes in the circulation by 55% whereas additional subcutaneous application of IL‐4 further increased the lifespan to 175% compared to vehicle‐treated WT monocytes in our experiments. To analyze if the observed IL‐4 effect is physiologically relevant in humans, we determined IL‐4 serum levels and monocyte counts in a human clinical cohort. The concentration of IL‐4 in serum was significantly associated with the amount of circulating monocytes. This leads us to hypothesize that the mechanisms identified in mice could also play a role in monocyte homeostasis in humans. However, further studies are needed to verify the data from this human cohort, as there are some limitations regarding the human cohort used here in our paper: for example, the subjects were cancer patients and newly diagnosed as well as recurrent cancer patients were included in this cohort.

IL‐4/IL‐13 signaling is associated with an anti‐inflammatory phenotype in monocytes and macrophages.^36^ Interestingly, we found that knockout of IL‐4Rα signaling in monocytes led to reduced levels of inflammatory cytokines in the circulation during steady state. As stimulation with IL‐4 did not change the transcription levels of these cytokines in murine and human monocytes, we conclude that the reduction in overall monocyte count resulted in diminished plasmatic levels of monocyte‐derived cytokines. Our data are in line with other studies, identifying monocytes as contributors to IL‐1α and MCP‐1 plasma levels during homeostasis.^37^, ^38^

The exact mechanisms ultimately regulating the fate and death of monocytes in circulation are not yet completely elucidated. Swirski et al. showed, that the spleen harbors a reservoir of monocytes, which can be recruited to inflamed sites.^27^ When we analyzed different tissues in IL‐4Rα knockout mice, we found the amount of monocytes in spleens of IL‐4Rα‐deficient mice to be increased. Furthermore, genes involved in apoptotic pathways in the transcriptome of these cells were altered and we were able to identify markers of increased cell death in the splenic monocyte reservoir, pointing toward an increased turnover of monocytes in mice lacking IL‐4 receptor signaling. This observation was supported by our in vitro experiments using human monocytes, showing that stimulation with IL‐4 effectively prevented apoptosis. Similar to our finding, IL‐4 was shown in vitro to prevent apoptosis in basophil granulocytes.^39^

Monocyte recruitment into tissues is a common and important step in the propagation and progression of many inflammatory diseases, as they are found for example in peritoneal lavage fluid of patients with ascites.^40^ By showing that IL‐4Rα is critically involved in monocyte survival, we identify this receptor as a promising target in such inflammatory disease states. It should be noted that Type II IL‐4 receptors are also expressed on non‐hematopoietic cells^41^ and that IL‐4 signaling was also described to be involved in regulating vascular endothelial permeability.^31^ Therefore, future studies are warranted to evaluate the potential of targeting IL‐4 receptors on monocytes to therapeutically modulate their pathogenic effects in inflammatory diseases.

## AUTHOR CONTRIBUTIONS

Patrick Haider, Philipp J. Hohensinner, and Johann Wojta were involved in conceptualization, methodology, and project administration. Patrick Haider and Florian Moik were involved in formal analysis. Patrick Haider, Julia B. Kral‐Pointner, Manuel Salzmann, Florian Moik, Sonja Bleichert, Christoph Kaun, and Mira Brekalo were involved in investigation. Patrick Haider was involved in visualization. Johann Wojta, Ingrid Pabinger, Christine Brostjan, and Sylvia Knapp were involved in funding acquisition. Philipp J. Hohensinner and Johann Wojta were involved in supervision. Patrick Haider and Philipp J. Hohensinner were involved in writing—original draft. Waltraud C. Schrottmaier, Walter S. Speidl, Bruno K. Podesser, Kurt Huber, Sylvia Knapp, Frank Brombacher, Christine Brostjan, Cihan Ay, and Johann Wojta were involved in writing—review and editing.

## DISCLOSURES

The authors have stated explicitly that there is no conflict of interest in connection with this article.

## Supporting information


Appendix S1

## Data Availability

The RNAseq data and corresponding metadata are available in the Gene Expression Omnibus (GEO) database of NCBI under the accession number GSE168143. All other data that support the findings of this study are available from the corresponding author on request.
