# Supplementary material for: Interleukin‐4 receptor alpha signaling regulates monocyte homeostasis
Source: FASEB J. 2022 Sep 5;36(10):e22532. doi: 10.1096/fj.202101672RR (PMC9544925; doi:10.1096/fj.202101672RR)
Supplement: Supplementary file 1 — Appendix S1 [file FSB2-36-0-s001.docx]

**Supplementary Information for**

Interleukin-4 receptor alpha signaling regulates monocyte homeostasis

Patrick Haider,^1^ Julia B. Kral-Pointner,^1,2^ Manuel Salzmann,^1^ Florian Moik,^3^ Sonja Bleichert,^4^ Waltraud C. Schrottmaier,^5^ Christoph Kaun,^1^ Mira Brekalo,^1^ Michael B. Fischer,^6,7^ Walter S. Speidl,^1^ Christian Hengstenberg,^1^ Bruno K. Podesser,^2,8^ Kurt Huber,^9,10^ Ingrid Pabinger,^3^ Sylvia Knapp,^11^ Frank Brombacher,^12^ Christine Brostjan,^4^ Cihan Ay,^3^ Johann Wojta,^1,2,13^ Philipp J. Hohensinner^1,2,8^

^1^Department of Internal Medicine II/Division of Cardiology, Medical University of Vienna, Vienna, Austria.
^2^Ludwig Boltzmann Institute for Cardiovascular Research, Medical University of Vienna, Vienna, Austria.
^3^Department of Internal Medicine I/Division of Haematology and Haemostaseology, Comprehensive Cancer Center, Medical University of Vienna, Vienna, Austria.
^4^Department of General Surgery/Division of Vascular Surgery, Medical University of Vienna, Vienna, Austria.
^5^Institute of Vascular Biology and Thrombosis Research, Medical University of Vienna, Vienna, Austria.
^6^Department of Blood Group Serology and Transfusion Medicine, Medical University of Vienna; Vienna, Austria.
^7^Department of Biomedical Research, Danube University Krems, Krems, Austria.
^8^Center for Biomedical Research, Medical University of Vienna, Vienna, Austria.
^9^3rd Department of Medicine, Cardiology and Intensive Care Medicine, Wilhelminenhospital, Vienna, Austria.
^10^Medical Faculty, Sigmund Freud University, Vienna, Austria.
^11^Department of Internal Medicine I/Laboratory of Infection Biology, Medical University of Vienna, Vienna, Austria.
^12^Institute of Infectious Disease and Molecular Medicine, International Center for Genetic and Biotechnology Cape Town Component & University of Cape Town, Cape Town, South Africa.
^13^Core Facilities, Medical University of Vienna, Vienna, Austria.


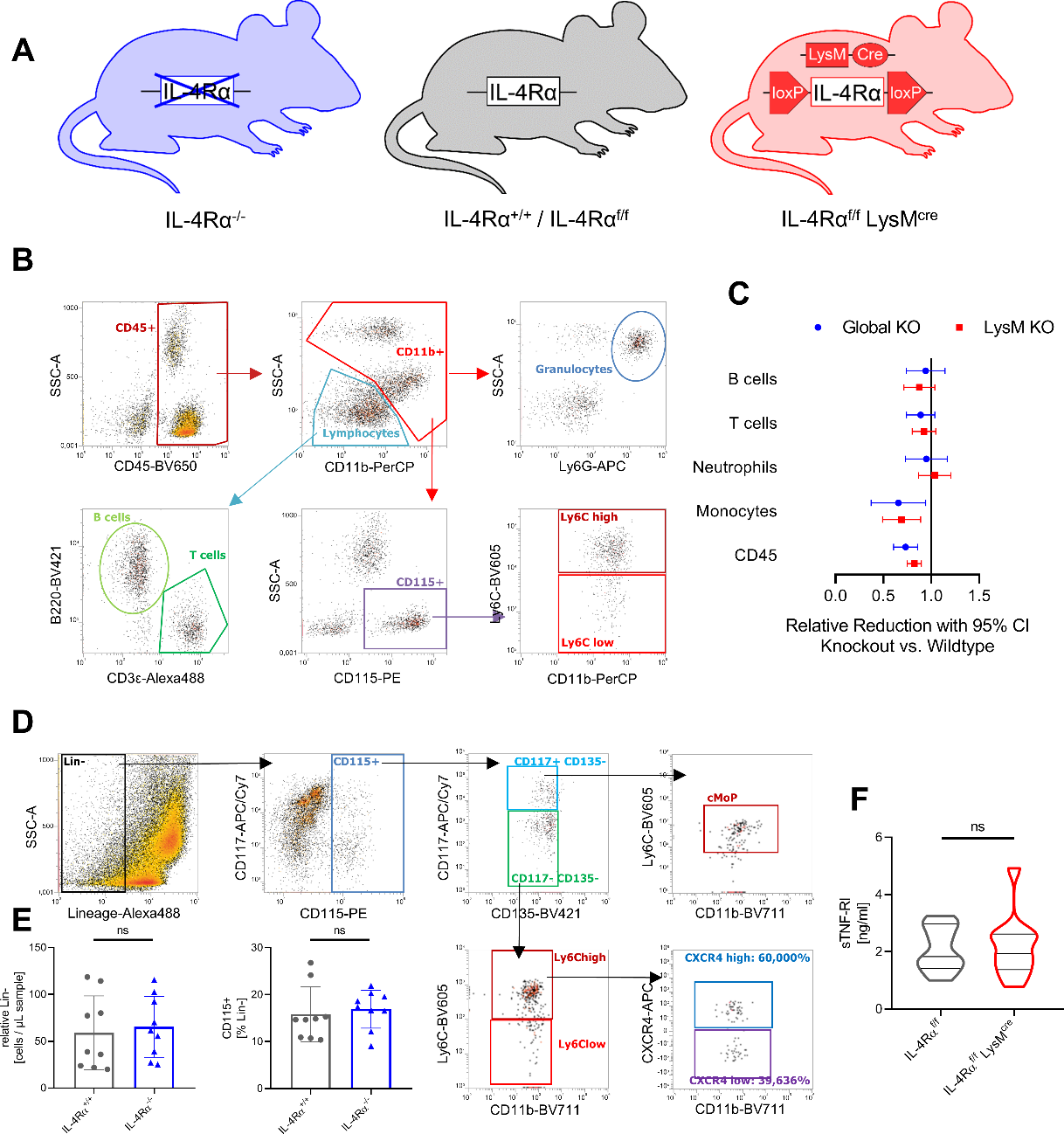


Supplemental Figure 1. Other circulating leukocyte populations are not reduced in IL-4Rα knockout mice. (A) Genetic scheme of mouse lines and respective controls. (B) Flow cytometric gating strategy for blood leukocyte populations. (C) Relative reduction of the different leukocyte populations with 95% confidence intervals in the blood of IL-4Rα^-/-^ and IL-4R^f/f^ LysM^cre^ from Figure 1 compared to their respective wildtype littermates. (D) Flow cytometric gating strategy for bone marrow monocytic lineage populations. (E) Flow cytometry analysis of relative amount of lineage- cells and CD115+ cells in the bone marrow of IL-4Rα^+/+^ and IL-4Rα^-/-^ mice (n = 9 per genotype). (F) Violin plot of sTNF-RI quantified in plasma of IL-4Rα^f/f^ and IL-4Rα^f/f^ LysM^cre^ mice (n = 18 per genotype, ns = not significant).


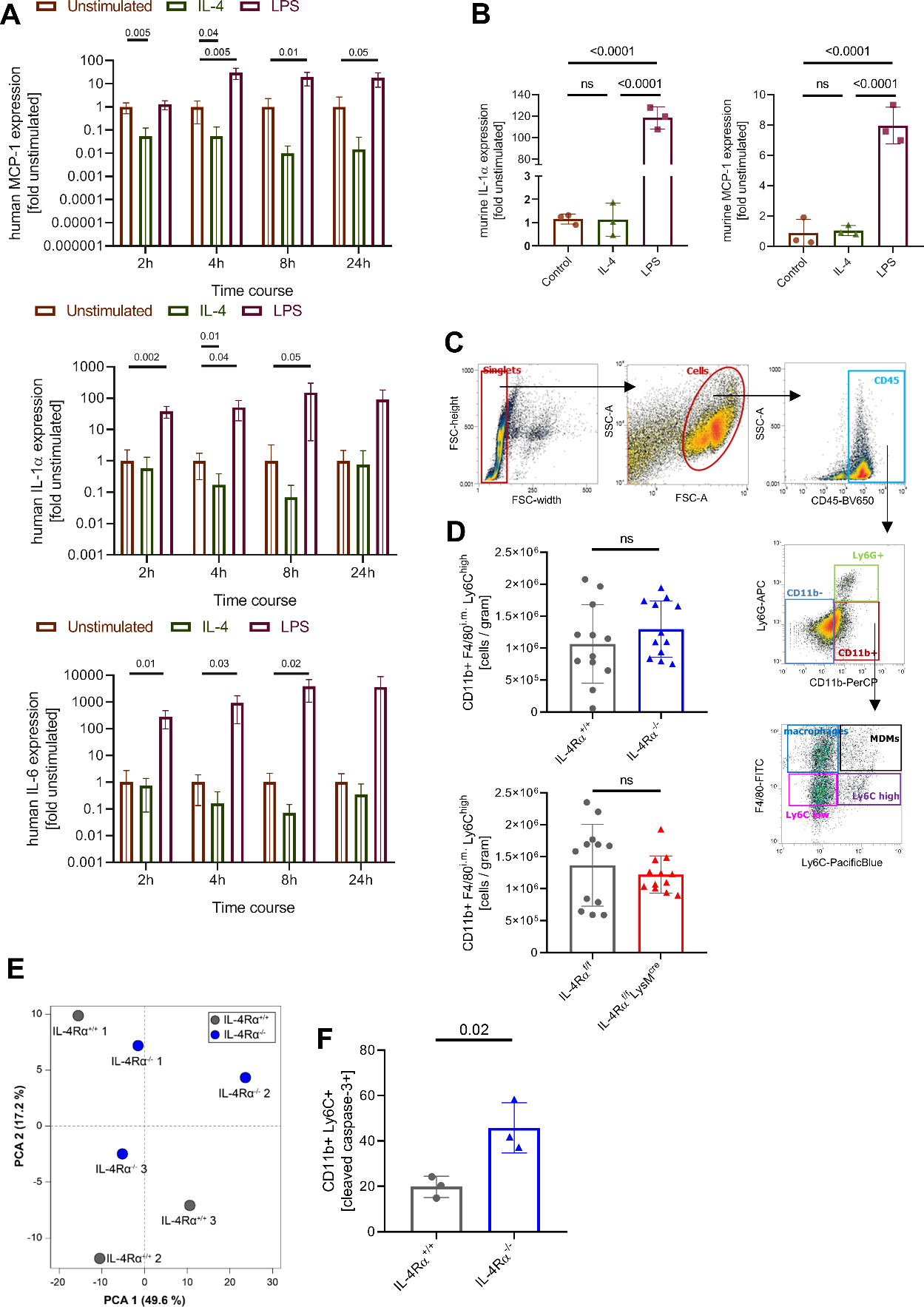


Supplemental Figure 2. Analysis of RNA expression in murine and human monocytes. (A) Time course of the expression of MCP-1, IL-1α and IL-6 mRNA measured by qPCR from isolated human monocytes either left unstimulated (Control) or stimulated for 2, 4, 8 or 24 hours with 100 ng/mL lipopolysaccharide (LPS) or IL-4 (n = 6 human donors). (B) Expression of IL-1α and MCP-1 mRNA measured by qPCR from isolated murine monocytes either left unstimulated (Control) or stimulated for 4 hours with 100 ng/mL lipopolysaccharide (LPS) or IL-4. (n = 3 C57BL/6J mice). (C) Flow cytometric gating strategy for splenic leukocyte populations. (D) Flow cytometry analysis of absolute numbers of CD11b^+^F4/80^i.m^Ly6C^high^ monocytes in the spleens of IL-4Rα^+/+^ and IL-4Rα^-/-^ mice (n = 12 per genotype) and IL-4Rα^f/f^ and IL-4Rα^f/f^ LysM^cre^ mice (n = 12 per genotype) (E) Plot of the first 2 dimensions of a principle component analysis, based on the reg_log_transf_counts data of isolated murine spleen monocytes from IL-4Rα^+/+^ and IL-4Rα^-/-^ mice. (n = 3 per genotype) (F) Flow cytometry analysis of cleaved caspase-3-positive CD11b^+^Ly6C^+^ monocytes in the spleens of IL-4Rα^+/+^ and IL-4Rα^-/-^ mice (n = 3 per genotype)


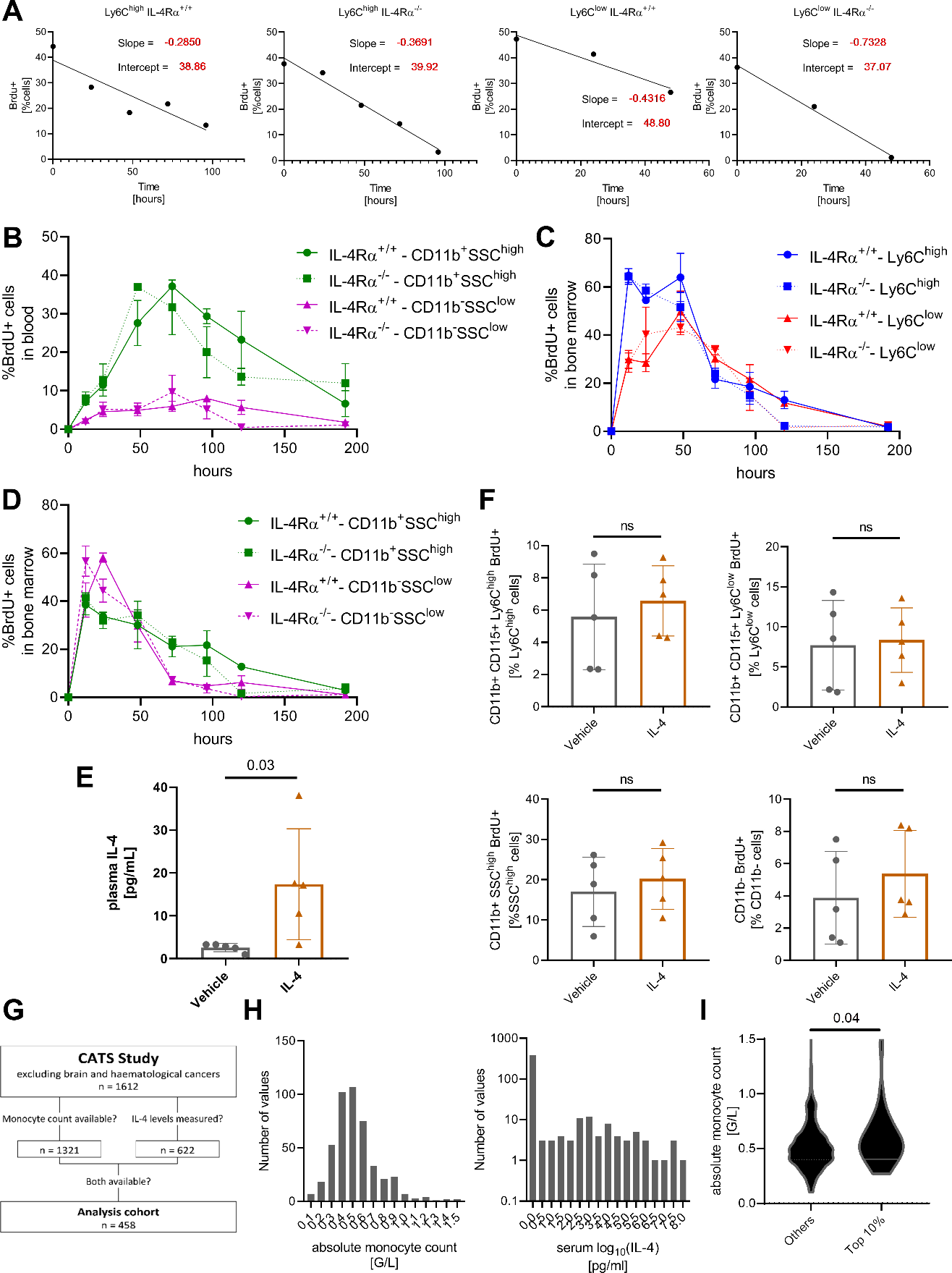


## Supplemental Figure 3. Knockout of IL-4Rα does not alter the lifespan of granulocytes or lymphocytes. (A) Linear regression for the slope curve of each monocyte subset and genotype. (n = 3 per genotype and time point) (B) Time kinetic graph of BrdU means ± s.e.m. in CD11b^+^SSC^high^ granulocytes and CD11b^-^SSC^low^ lymphocytes in the blood. (n = 3 per genotype and time point) (C and D) Time kinetic graph of BrdU means ± s.e.m. in monocyte subsets (C) as well as CD11b^+^SSC^high^ granulocytes and CD11b^-^SSC^low^ lymphocytes (D) in the bone marrow. (n = 3 per genotype and time point) (E) IL-4 quantified by ELISA in plasma of C57BL/6J mice. (n = 5 mice per group) (F) Flow cytometry analysis of BrdU signal in CD11b^+^CD115^+^ monocyte subsets as well as CD11b^+^SSC^high^ granulocytes and CD11b^-^SSC^low^ lymphocytes in the bone marrow of C57BL/6J mice. (n = 5 mice per group) (G) Flow diagram for patient inclusion from the Vienna CATS study. (H) Distribution of measured absolute monocyte counts (left) and IL-4 levels (right) in the analysis cohort (n = 458) patients. (I) Violin plot of absolute monocyte counts in the analysis cohort (n = 458), stratified by the amount of IL-4 per patient.


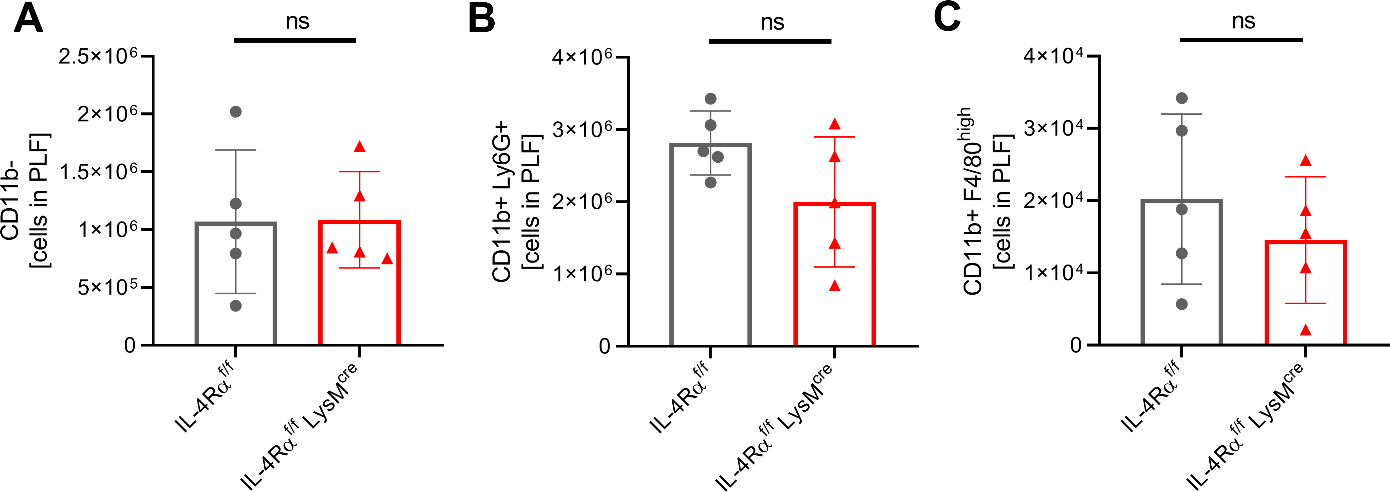


Supplemental Figure 4. Other leukocyte populations are not affected in sterile peritonitis model. (A - C) Flow cytometry analysis of absolute numbers of CD11b- lymphocytes (A), CD11b^+^Ly6G^+^ granulocytes (B) and CD11b^+^F4/80^high^ macrophages (C) in the peritoneal lavage fluid of IL-4Rα^f/f^ and IL-4Rα^f/f^ LysM^cre^ mice 12 hours after thioglycollate injection. (n = 5 mice per group)

Supplementary tables

Supplemental Table 1. Overview of the respective relative means of fold change and significances of the cytokine array in Figure 2. Bold p values indicate significantly different cytokines identified by t-test following post-hoc analysis for multiple testing.

| **Cytokine Array Data** | | **Analysis cohort (n = 4 per genotype)** | |
| --- | --- | --- | --- |
|  |  |  |  |
| **Cytokine** | | **Log2(fold)** | **-Log(p)** |
| IL_6 | | 0.206839 | 0.142903 |
| CD30L | | 0.195377 | 0.437838 |
| Eotaxin_2 | | 0.061966 | 0.081838 |
| IL_4 | | 0.011198 | 0.022175 |
| IL_12_p40_p70 | | 0.001939 | 0.003984 |
| IL_9 | | -0.02353 | 0.033807 |
| GM_CSF | | -0.03146 | 0.0386 |
| IL_7 | | -0.04045 | 0.030511 |
| IL_10 | | -0.04755 | 0.024635 |
| Eoxtaxin | | -0.05544 | 0.036571 |
| MIG | | -0.06173 | 0.028749 |
| MIP_1a | | -0.07306 | 0.053063 |
| IL_12_p70 | | -0.10254 | 0.087059 |
| LIX | | -0.11744 | 0.229894 |
| MIP_1y | | -0.13499 | 0.365144 |
| KC | | -0.16929 | 0.473614 |
| IL_2 | | -0.17731 | 0.173105 |
| RANTES | | -0.18298 | 0.1964 |
| TCA_3 | | -0.18637 | 0.467502 |
| Lymphotactin | | -0.19963 | 0.943034 |
| IFNy | | -0.20696 | 0.317618 |
| BLC | | -0.20751 | 0.908301 |
| IL_13 | | -0.23804 | 0.755738 |
| MCSF | | -0.23968 | 0.748513 |
| I_TAC | | -0.27366 | 0.326375 |
| sTNF_RII | | -0.28765 | 0.594948 |
| FasL | | -0.31195 | 0.270723 |
| TIMP_1 | | -0.31887 | 0.279336 |
| TECK | | -0.32163 | 0.478095 |
| Fractalkine | | -0.32897 | 0.523657 |
| MCP_1 | | -0.33133 | **2.137451** |
| IL_1a | | -0.37274 | **1.769092** |
| Leptin | | -0.38966 | 0.357483 |
| SDF_1 | | -0.42141 | 0.748698 |
| IL_1b | | -0.49923 | 0.720164 |
| TIMP_2 | | -0.55594 | 0.744909 |
| TNFa | | -0.57149 | 1.268508 |
| IL_17 | | -0.61135 | 0.931896 |
| sTNF_RI | | -0.66659 | **4.275724** |
| GCSF | | -0.79057 | 0.578269 |

Supplemental Table 2. Overview of the respective relative means of fold change and significances of the phosphorylated apoptosis pathway array in Figure 5. Bold p values indicate significantly different proteins identified by t-test following post-hoc analysis for multiple testing.

| **Phosphorylation Apoptosis Pathway**  **Array Data** | | **Analysis cohort (n = 4 per condition)** | |
| --- | --- | --- | --- |
|  |  |  |  |
| **Protein target** | | **Log2(fold)** | **-Log(p)** |
| IκBα (Ser32) | | 0.697973 | **1.653647** |
| TAK1 (Ser412) | | 0.642441 | **2.455932** |
| BAD (Ser112) | | 0.618702 | **1.970616** |
| JNK (Thr183/Tyr185) | | 0.372262 | **2.481486** |
| NFκBp65 (Ser536) | | 0.335656 | **1.308919** |
| HSP27 (Ser82) | | 0.323488 | 0.169925 |
| CD30L | | 0.195377 | 0.437838 |
| p38 (Thr180/Tyr182) | | 0.160431 | 0.541211 |
| Caspase7 (cleaved Asp198) | | 0.100204 | 0.492279 |
| ERK1/2 (T202/Y204 / Y185/Y187) | | 0.082265 | 0.06839 |
| SMAD2 (Ser245/250/255) | | 0.069837 | 0.06083 |
| Eotaxin_2 | | 0.061966 | 0.081838 |
| eIF2a (Ser51) | | 0.045265 | 0.025534 |
| p27 (Thr198) | | 0.036762 | 0.168386 |
| CHK2 (Thr68) | | 0.023094 | 0.112439 |
| ATM (Ser1981) | | 0.000811 | 0.03574 |
| p53 (Ser15) | | -0.01491 | 0.123609 |
| GM_CSF | | -0.03146 | 0.0386 |
| AKT (Ser473) | | -0.03847 | 0.190844 |
| Eoxtaxin | | -0.05544 | 0.036571 |
| Caspase3 (cleaved Asp175) | | -0.08104 | 0.330033 |
| CHK1 (Ser296) | | -0.08319 | 0.343231 |
| IFNy | | -0.20696 | 0.317618 |
| BLC | | -0.20751 | 0.908301 |
| PARP1 (cleaved Asp214/Gly215) | | -0.26512 | 0.900319 |
| FasL | | -0.31195 | 0.270723 |
| Fractalkine | | -0.32897 | 0.523657 |
| GCSF | | -0.79057 | 0.578269 |

Supplemental Table 3. Patient demographics.

| **Demographics CATS study** | | **Analysis cohort (n = 458)** | |
| --- | --- | --- | --- |
|  |  |  |  |
| **Metric variables** | | **Average** [Unit] | **Dispersion** [Unit] |
| Age, years | | 63 [median] | 56-69 [IQR] |
| Absolute monocyte count, % | | 0.53 [mean] | 0.22 [SD] |
| IL-4, ng/ml | | 31.2 [mean] | 207.6 [SD] |
|  |  |  |  |
| **Categorical variables** | |  | **Count (%)** |
| Sex |  |  |  |
|  | Female |  | 215 (47%) |
|  | Male |  | 243 (53%) |
| Tumor type |  |  |  |
|  | Breast |  | 106 (23%) |
|  | Colon |  | 83 (18%) |
|  | Prostate |  | 80 (17%) |
|  | Lung |  | 77 (17%) |
|  | Pancreas |  | 40 (9%) |
|  | Gastric |  | 24 (5%) |
|  | Renal |  | 18 (4%) |
|  | Other |  | 30 (7%) |
| Diagnosis |  |  |  |
|  | Newly diagnosed |  | 343 (75%) |
|  | Recurrent cancer |  | 115 (25%) |
| Metastatic cancer |  |  |  |
|  | Yes (i.e. Stage IV) |  | 219 (48%) |
|  | No |  | 237 (52%) |

Supplemental Table 4. Statistical analysis of subgroups stratified by IL-4 amounts.

| **Subgroup analysis** | | **Overall** | **Low** | **High** |  |
| --- | --- | --- | --- | --- | --- |
|  |  | **n = 69** | **n = 35** | **n = 34** | **p-value** |
|  |  |  |  |  |  |
| Age [mean (SD)] | | 64 (12) | 62 (10) | 65 (13) | 0.362 |
|  |  |  |  |  |  |
| Sex |  |  |  |  |  |
|  | Female | 49% | 47% | 53% | 0.717 |
|  | Male | 51% | 53% | 47% | / |
|  |  |  |  |  |  |
| Tumor type | | / | / | / | 0.295 |
|  | Breast | 17% | 12% | 23% | / |
|  | Colon | 25% | 26% | 23% | / |
|  | Prostate | 17% | 21% | 14% | / |
|  | Lung | 14% | 18% | 11% | / |
|  | Pancreas | 12% | 6% | 17% | / |
|  | Gastric | 4% | 8% | 0% | / |
|  | Renal | 4% | 6% | 3% | / |
|  | Other | 6% | 3% | 9% | / |
|  |  |  |  |  |  |
| Diagnosis | |  |  |  |  |
|  | Newly diagnosed | 70% | 71% | 69% | 0.856 |
|  | Recurrent cancer | 30% | 29% | 31% | / |
|  |  |  |  |  |  |
| Metastatic cancer | |  |  |  |  |
|  | Yes (i.e. Stage IV) | 53% | 53% | 54% | 0.911 |
|  | No | 47% | 47% | 46% | / |
